# Comparative genomics of Balto, a famous historic dog, captures lost diversity of 1920s sled dogs

**DOI:** 10.1126/science.abn5887

**Published:** 2023-04-28

**Authors:** Katherine L. Moon, Heather J. Huson, Kathleen Morrill, Ming-Shan Wang, Xue Li, Krishnamoorthy Srikanth, Zoonomia Consortium, Kerstin Lindblad-Toh, Gavin J. Svenson, Elinor K. Karlsson, Beth Shapiro

**Affiliations:** 1 Department of Ecology and Evolutionary Biology, University of California Santa Cruz, Santa Cruz, CA, USA; 2 Howard Hughes Medical Institute, University of California Santa Cruz, Santa Cruz, CA, USA; 3 Department of Animal Sciences, Cornell University College of Agriculture and Life Sciences, Ithaca, NY, 14853, USA; 4 Bioinformatics and Integrative Biology, UMass Chan Medical School, Worcester, MA 01655, USA; 5 Morningside Graduate School of Biomedical Sciences, UMass Chan Medical School, Worcester, MA 01655, USA; 6 Broad Institute of MIT and Harvard, Cambridge, MA 02142, USA; 7 Department of Medical Biochemistry and Microbiology, Science for Life Laboratory, Uppsala University; Uppsala, 751 32, Sweden.; 8 Cleveland Museum of Natural History, Cleveland, OH 44106, USA

## Abstract

We reconstruct the phenotype of Balto, the heroic sled dog renowned for transporting diphtheria antitoxin to Nome, Alaska in 1925, using evolutionary constraint estimates from the Zoonomia alignment of 240 mammals and 682 genomes from dogs and wolves of the 21st century. Balto shares just part of his diverse ancestry with the eponymous Siberian husky breed. Balto’s genotype predicts a combination of coat features atypical for modern sled dog breeds, and a slightly smaller stature. He had enhanced starch digestion compared with Greenland sled dogs and a compendium of derived homozygous coding variants at constrained positions in genes connected to bone and skin development. We propose that Balto’s population of origin, which was less inbred and genetically healthier than modern breeds, was adapted to the extreme environment of 1920s Alaska.

## One-Sentence Summary

Comparative genomics uncovers genotype-phenotype links between Balto, famed sled dog of the 1925 Serum Run, and modern dogs.

Technological advances in the recovery of ancient DNA make it possible to generate high-coverage nuclear genomes from historic and fossil specimens, but interpreting genetic data from past individuals is difficult without data from their contemporaries. Comparative genomic analysis offers a solution: by combining population-level genomic data and catalogs of trait associations in modern populations, we can infer the genetic and phenotypic features of long-dead individuals and the populations from which they were born. Zoonomia is a new comparative resource that addresses limitations of previous datasets (1) to support interpretation of paleogenomics data. With 240 placental mammal species, Zoonomia has sufficient power to distinguish individual bases under evolutionary constraint - a useful predictor of functional importance (2) - in coding and regulatory elements (3). Zoonomia’s reference-free genome alignment (4, 5) allows evolutionary constraint to be scored in any of its 240 species, including dogs.

Here, we generate a genome for Balto, the famous sled dog who delivered diphtheria serum to the children of Nome, Alaska, during a 1925 outbreak. Following his death, Balto was taxidermied and his remains are held by the Cleveland Museum of Natural History. We generated a 40.4-fold coverage nuclear genome from Balto’s underbelly skin using protocols for degraded samples. His DNA was well preserved, with an average endogenous content of 87.7% in sequencing libraries, low (<1%) damage rates (fig. S1) and short (68bp) average fragment sizes, consistent with the age of the sample.

Balto was born in the kennel of sled dog breeder Leonard Seppala in 1919. Although Seppala’s small fast dogs were known as Siberian huskies (6), they were a working population that differed from the dog breed recognized by the American Kennel Club (AKC) today. Modern dog breeds are genetically closed populations that conform to a tightly delineated physical standard (7). Balto’s relationship to AKC-recognized sled dog breeds like the Siberian husky (established in 1930) and Alaskan malamute (1935) (8) is unclear. Balto himself was neutered at six months of age and had no offspring.

Working populations of sled dogs survive. Alaskan sled dogs are bred solely for physical performance, including outcrossing with various breeds (9). Greenland sled dogs are an indigenous land-race breed that have been used for hunting and sledging by Inuit in Greenland for 850 years, where they have been isolated from contact with other dogs (10). Here, we use the term “breed” exclusively to refer to modern breeds recognized by the AKC or other kennel clubs (e.g. sled dog breeds), as distinct from the less rigidly defined populations of Greenland sled dogs and Alaskan sled dogs (working sled dogs). This is a genetic distinction; AKC-registered dogs can be successful working sled dogs.

We compared Balto to working sled dogs, sled dog breeds, other breeds, village dogs (free-breeding dogs without known breed ancestry), and other canids. Our whole genome dataset comprised 688 dogs (table S1) representing 135 breeds/populations, including three Alaskan sled dogs and five Greenland sled dogs (10). We identified evolutionarily constrained bases using phyloP evolutionary constraint scores from the dog-referenced version of the 240 species Zoonomia alignment (3).

**Figure 1.**
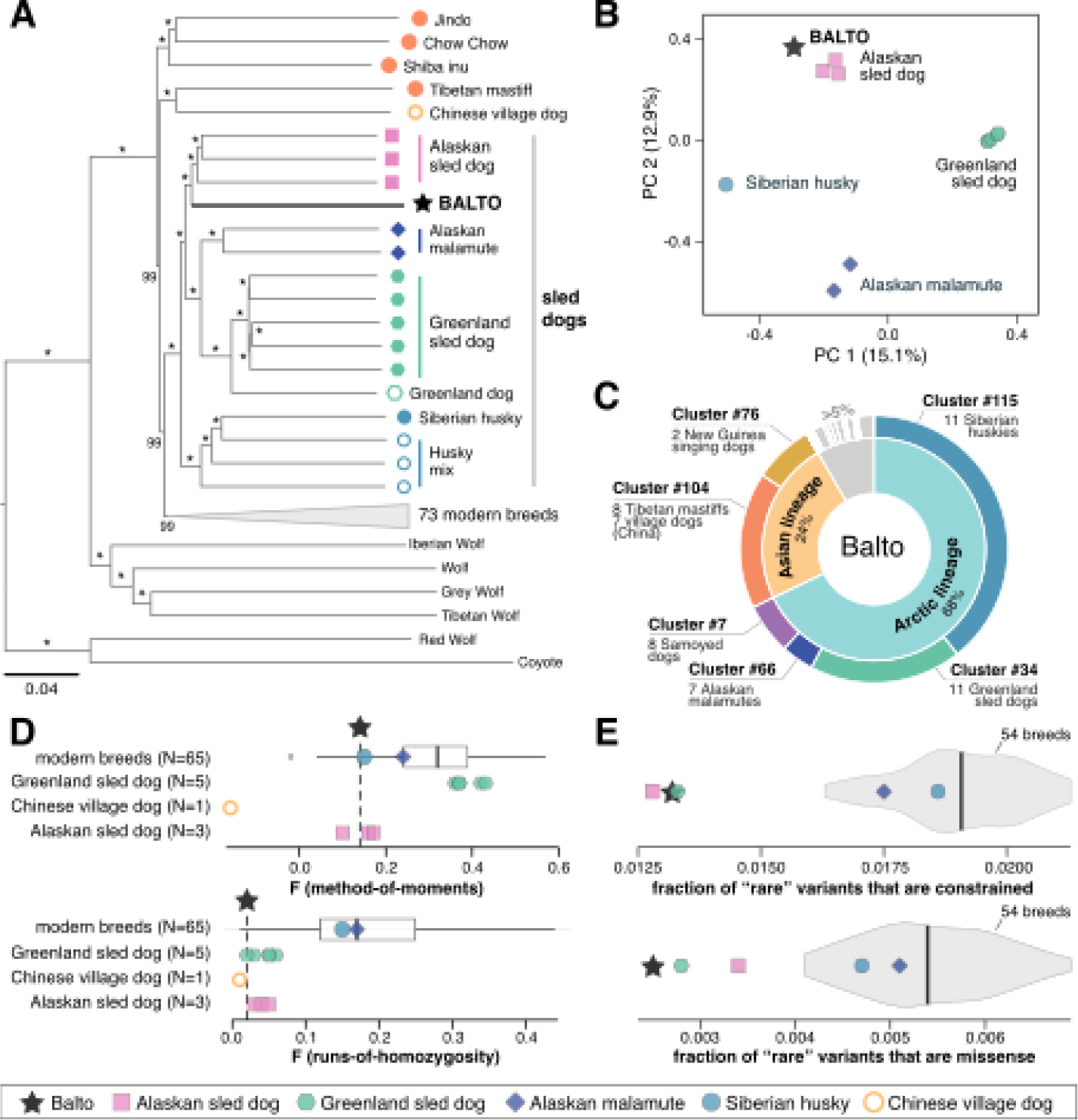
Balto clusters most closely with Alaskan sled dogs, but had high genetic diversity and a lower burden of potentially damaging variants. (**A**) Neighbor-joining tree clusters Balto (★) most closely with the outbred, working population of Alaskan sled dogs, and a part of a clade of sled dog populations. (**B**) Similarly, principal component analysis puts Balto near, but not in, a cluster of Alaskan sled dogs. (**C**) Unsupervised admixture analysis of Balto alongside the Alaskan sled dogs and other dogs and canids (*K*= 116 putative populations and N= 2166 individuals) infers substantial ancestral similarity to Siberian huskies, Greenland sled dogs, and outbred dogs from Asia (table S2). The remainder of his ancestry (8%) matches poorly (<5%) to any other clusters. Balto and working sled dogs (**D**) had lower levels of inbreeding, and (**E**) carried fewer constrained (*p*_wilcox_=0.0019) and missense (*p*_wilcox_= 0.0023) rare variants than modern dog breeds (table S10).

Ancestry analysis places Balto in a clade of sled dog breeds and working sled dogs and closest to the Alaskan sled dogs (Fig. 1A,B). Most of his ancestry is assigned to clades of Arctic-origin dogs (68%) and, to a lesser extent, Asian-origin dogs (24%) in an unsupervised admixture analysis with 2166 dogs and 116 clusters (Fig. 1C, table S2, S3). He carried no discernible wolf ancestry. The more recently established Alaskan sled dog population (9) did not fall into a distinct ancestry cluster in the unsupervised analysis, but comprised 34% of Balto’s ancestry in a supervised analysis defining them as a cluster (fig. S2).

Balto was more genetically diverse than breed dogs today and similar to working sled dogs (Fig. 1D). Balto had shorter runs of homozygosity than any breed dog, and fewer runs of homozygosity than all but one Tibetan mastiff (table S4). When inbreeding is calculated from runs of homozygosity, Balto and the two working sled dog populations are lower than almost any breed dog (fig. S3). When inbreeding is calculated using an allele frequency approach (method-of-moment), Greenland sled dogs have high inbreeding coefficients, reflecting their long genetic isolation in Greenland (fig. S3).

To evaluate the genetic health of Balto’s population of origin, we developed an analytical approach that leveraged the Zoonomia 240 species constraint scores and required only a single dog from each population (necessary since Balto is the only available representative of his population). Briefly, we selected one individual at random from each breed or population (57 dogs total) and scored variant positions as either evolutionarily constrained (and more likely to be damaging (2)) or not using the Zoonomia phyloP scores (3). We also identified variants likely to be “rare” (low frequency) in each dog’s breed or population. Because we couldn’t directly measure population allele frequencies with only a single representative dog, we defined “rare” variants as heterozygous or homozygous variants unique to that dog among all 57 representative dogs. This metric effectively identifies variants occurring at unusually low frequencies (fig. S4).

Balto and modern working sled dogs had a lower burden of rare, potentially damaging variation, indicating they represent genetically healthier populations (11) than breed dogs. Balto and the working sled dogs had significantly fewer potentially damaging variants (missense or constrained) than any breed dog, including the sled dog breeds (Fig. 1E). The pattern persists even in the less genetically diverse Greenland sled dog. Selection for fitness in working sled dog populations appears more effective in removing damaging genetic variation than selection to meet a breed standard.

**Figure 2.**
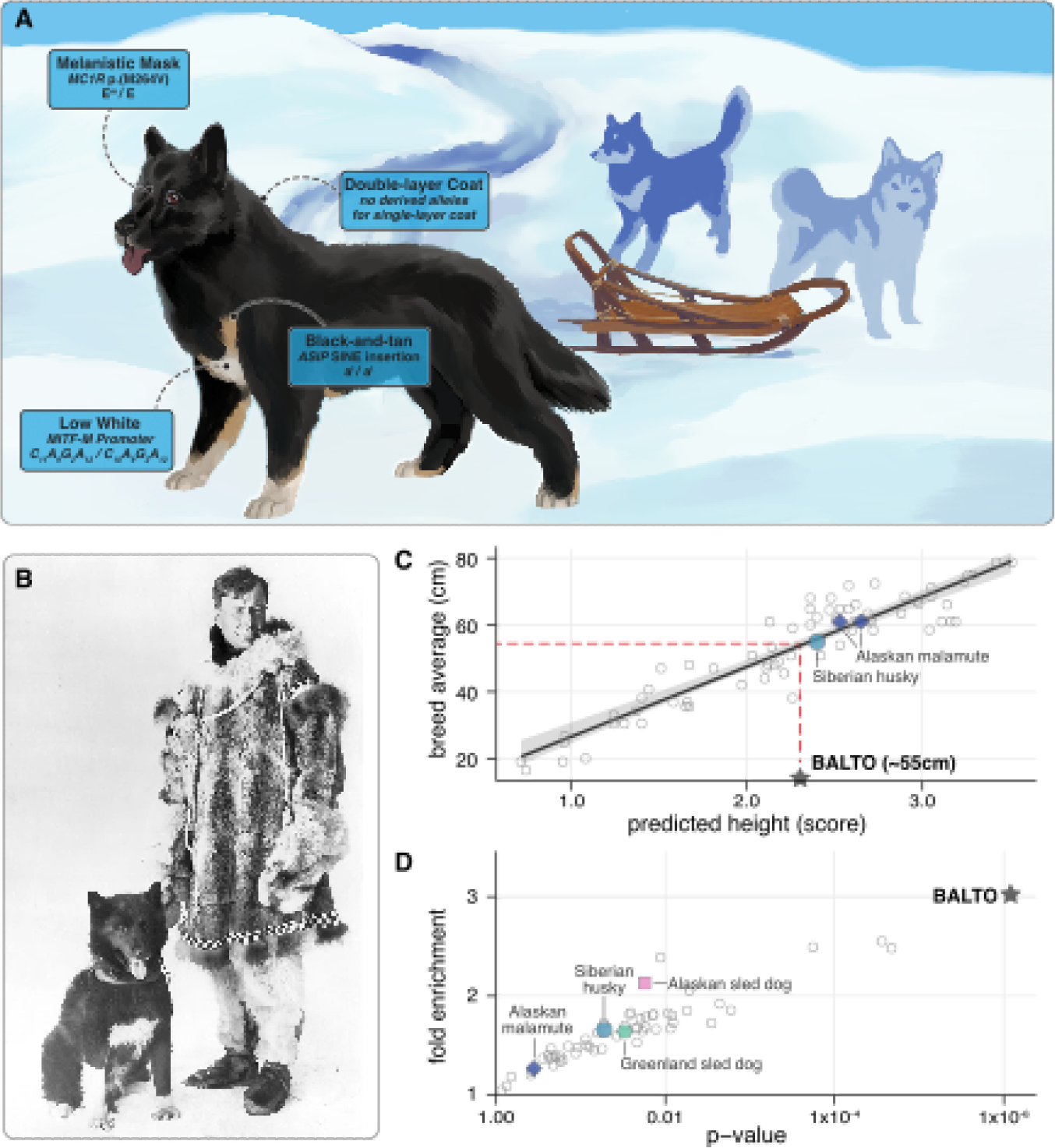
Genomic recreation of Balto’s physical appearance. (**A**) Prediction of Balto’s coat features based on his genome sequence with details on each trait and genotype in blue boxes. (**B**) A photo of Balto with musher Gunnar Kaasen. From the photo and his taxidermied remains, Balto was a black dog with dark eyes and some white patches on his chest and legs. He had a double-layered coat, and stood just under knee-high relative to Kaasen. Photo credit: Cleveland Museum of Natural History. (**C**) Using a random forest model based on 1,730 dogs and 2,797 height-associated genetic variants (12), we predicted that Balto would stand around 55 cm tall (value: 2.3) at his withers, close to the average height for the Siberian husky breed. Circles show dogs from other breeds. (**D**) Gene set enrichment testing of genes with common and constrained missense variants in 57 different dog populations shows a significant enrichment (p_FDR_=0.013) in the GO Tissue Development pathway only for Balto’s population.

Balto’s physical appearance predicted from his genome sequence (Fig. 2A, table S5) matches historical photos (Fig. 2B) and his taxidermied remains, indicating that the same variants shaping modern breed phenotypes also explained natural variation in his pre-breed working population. We predict that he stood 55cm tall at his shoulders (12)(Fig. 2C), within the acceptable range for today’s Siberian husky breed (53–60cm (8)), and had a double layered coat (13) that was mostly black with only a little bit of white (14). He was homozygous for an allele conferring tan points (15) and one for blue eyes (16), but both were masked by his melanistic facial mask (17), and his predicted light-tan pigmentation (18) may have been indistinguishable from white. He carried neither the “wolf agouti” nor “Northern domino” patterns that are common in the Siberian husky and other sled dog breeds today (19).

Both Balto and Alaskan sled dogs had unexpected evidence of adaptation to starch-rich diets. They carry the dog version of *MGAM*, a gene involved in starch processing that is differentiated between dogs and wolves (20) and one of fourteen regions analyzed for evidence of selective pressure in Balto’s lineage using a gene tree analysis (table S6). In earlier work, the high frequency of the wolf version of *MGAM* in Greenland sled dogs prompted speculation that reduced starch digestion might be a working sled dog trait (10). Our findings suggest this phenomenon is specific to Greenland sled dogs. Gene tree analysis places one of Balto’s chromosomes in the ancestral wolf cluster, and one to the derived dog cluster (fig. S5). Most Alaskan sled dogs carry the dog version (frequency=0.83). However, read coverage of the gene *AMY2B* suggests Balto had fewer copies of this gene than many modern dogs, and thus comparatively lower production of the starch-digesting enzyme amylase (21, 22). Taken together, we suggest Balto’s ability to digest starch was enhanced compared to wolves and Greenland sled dogs, but reduced compared to modern breeds.

Of the other 14 regions tested, most (10/14) lacked sufficient diversity in dogs to resolve phylogenetic relationships. Bootstrap support was weak for two other genes selected in Greenland sled dogs (*CACNA1A* and *MAGI2*). As expected, Balto did not carry versions of *EPAS1* associated with high altitude adaptation (23).

We found an enrichment for unusual function variation in Balto’s population consistent with adaptation to the extreme environments in which early 20th century sled dogs worked. We identified variants in Balto’s genome that were new (not seen in wolves) and likely to be common in his population (homozygous in Balto; fig. S4). We further filtered for variants that were both protein-altering (missense) and evolutionarily constrained (FDR<0.01), and thus likely to be functional. Balto was no more likely to carry such variants than dogs from 54 other populations (fig. S6), but in Balto these variants tended to disrupt tissue development genes (GO:0009888; 24 genes; 3.02-fold enrichment; *p*_FDR_=0.013)(table S7). This enrichment was unique to Balto (Fig. 2D, fig. S7), and most of the variants were rare or missing in other dog populations (fig. S8). Even when all GO biological process gene sets are tested in all 57 dogs, Balto’s enrichment in tissue development genes is highly unusual. It ranks 4th out of 888,573 dog/set pairs tested (fig. S7, table S8). Phenotype associations from human disease studies suggest that these variants could have influenced skeletal and epithelial development including joint formation, body weight, coordination, and skin thickness (table S9)(24). Modern sled dog breeds and working sled dogs are only slightly more similar to Balto than other dogs at these variants (fig. S9).

Balto was part of a famed population of small, fast, and fit sled dogs imported from Siberia. Following his famous run, the Siberian husky breed was recognized by the AKC. By sequencing his genome from his taxidermied remains and analyzing it in the context of large comparative and canine datasets, we show that Balto shared only part of his ancestry with today’s Siberian huskies. Balto’s working sled dog contemporaries were healthier and more genetically diverse than modern breeds, and may have carried variants that helped them survive the harsh conditions of 1920s Alaska (6). Further work is still needed to assess the impact of the evolutionarily constrained missense variants that Balto carried. While the era of Balto and his fellow huskies has passed, comparative genomics, supported by a growing collection of modern and past genomes, can provide a snapshot of individuals and populations from the past, as well as insights into the selective pressures that shaped them.

## Materials and Methods:

### Assembly of comparative canid genetic variants

We collated a reference set of comparative canid genetic variants starting from the curated Broad-UMass Canid Variant set (https://data.broadinstitute.org/DogData/) and comprising whole genome sequencing data for 531 dogs of known breed ancestry distributed among 132 breeds, 28 dogs of mixed breed ancestry, 12 dogs of unknown ancestry, 69 worldwide indigenous or village dogs, 33 wolves, and 1 coyote (see stable S1).

### Ancient DNA extraction, library preparation, and genome assembly

We extracted DNA from a ~5mm × 5mm piece of Balto’s underbelly skin tissue, in two replicates (HM246 and HM247) with an extraction negative, using the ancient DNA specific protocol in Dabney et al. 2013 (28). We prepared 32 ~1pmol input Illumina libraries from these extracts following the Santa Cruz library preparation method (29), including positive and negative controls. All 32 libraries passed quality control (QC), and so we sequenced them to a depth of ~2.3 billion on a NovaSeq 6000 platform 150bp paired end (see table S11 for the number of reads produced per library).

We used SeqPrep v.1.1 (30) to trim adapters, remove reads shorter than 28bp, and merge remaining paired-end reads with a minimum overlap of 15 bp. We then used the Burrows-Wheeler Aligner (BWA) v.0.7.12 (31) with a minimum quality cut off of 20 to align reads to the *Canis lupus familiaris* (dog) reference genome (CanFam3.1) (NCBI: GCA_000002285.2). All 32 bam files (one for each library) were merged into one with PCR duplicates removed. We used both Qualimap (v2.2.1) and samtools (v1.7) to calculate metrics and assess the quality of the alignment (see table S12).

### Variant calling

We used GATK HaplotypeCaller to call variants in Balto as well as 10 previously published Greenland sled dogs (10) and 3 Alaskan sled dogs sequenced for this study (see Supplementary Methods for details on sampling, DNA extraction, and sequencing) against the UMass-Broad Canid Variant set using parameter *--genotyping-mode* GENOTYPE_GIVEN_ALLELES *--alleles* (known alleles). Then, we merged variant call records from these 14 dogs with records from the UMass-Broad Candid Variants set, for variant calls in a full set of 688 individuals: Balto (this study), 3 modern Alaskan sled dogs (this study), 10 modern Greenland sled dogs (10), 531 dogs from modern breeds, 40 dogs of unknown or admixed ancestry, 69 village or indigenous dogs, 33 wolves, and 1 coyote.

### Phylogenetic analysis and neighbor-joining trees

Using a dataset of 100 representative canids (see table S1 for samples selected in the `Phylogenetic Analysis`) we confirmed Balto’s phylogenetic position by generating a neighbor-joining (NJ) phylogenetic tree and conducting a principal component analysis (PCA). We converted the variant calls into a FASTA file and used MEGA-CC(33) with 1000 bootstraps to assess tree topology. We also ran a PCA on this set using *PLINK* (v1.9), and then visualized the first two principal components in R (v. 3.6.3) using the `ggplot2` package.

### Global ancestry inference

We inferred Balto’s ancestral similarity to modern dog breeds, sled dog type breeds, and working sled dogs using a custom built reference panel of modern dogs and canids of the 21st century (table S3). In *PLINK* (v2.00a3LM) (35), we identified 4,267,732 biallelic single nucleotide polymorphisms with <10% missing genotypes, and calculated Wright’s F-statistics using Hudson method (36, 37) for (1) each dog breed and sled dog population versus all other dogs; (2) all village dogs versus all other dogs; (3) each regional village dog population; (4) all wolves versus all other dogs; (5) all coyotes versus all other canids; and (6) North American wolves versus Eurasian wolves. We selected 1,858,634 SNPs with *F*_*ST*_>0.5 across all comparisons, and performed LD-based pruning in 250kb windows for *r*^*2*^>0.2 to extract 136,779 markers for global ancestry inference. We merged Balto’s genotypes for these SNPs with genotypes from the reference samples. For reference samples also represented in the whole genome dataset, population labels used in the admixture analysis are given in the `Representative in Global Ancestry Inferencè column of table S1. We performed global ancestry inference using *ADMIXTURE (38)* in both supervised mode (random seed: 43) with 20 bootstrap replicates to estimate parameter standard errors, and in unsupervised mode for the same number of populations (*K*=116), which showed low levels of error (0.3) in ten-fold cross-validation analysis of chromosome 1 for *K* clusters between 50 and 150 (table S13).

### Homozygosity and inbreeding metrics

We removed samples with any missing data from the dataset of 100 representative individuals used in the phylogenetic analyses, leaving 86 individuals (see table S1 for samples selected in the ‘Homozygosity Analysis’). Using this pruned dataset, we detected runs of homozygosity (RoH) using a window-based approach implemented in *PLINK* (v1.9) (35). We calculated two measures of inbreeding: the method-of-moments coefficient in *PLINK* (*F*_*MoM*_) and the metric based on runs-of-homozygosity (*F*_*RoH*_), as recommended by Zhao et al. 2020 (40) (table S4). Using the *R* (v. 3.6.3) function `cor.test`, we confirmed that *F*_*RoH*_ and *F*_*MoM*_ are significantly correlated (*R*_Pearson_= 0.6752819, *p*= 9.958e-13, *t*= 8.3913, *df*= 84).

### Population representative sampling

As Balto is the sole representative of his population, we randomly selected one representative sample from each of 57 populations for the discovery of individually-represented, population-relevant genetic variants (see table S1 for samples selected in the `Population Variants Analysis`) among 67,085,518 biallelic single nucleotide polymorphisms. These populations included Balto, 1 Alaskan sled dog, 1 Greenland sled dog, and 54 modern purebred dogs, including 1 Siberian husky and 1 Alaskan malamute. Likewise, we selected, where available, another 5 to 11 random samples from 10 modern breeds, and all remaining Greenland sled dog samples, to assess the population-wide allele frequency of these variants (see table S1 `Population Frequency Analysis`).

### Dog-referenced mammalian evolutionary constraint

We selected biallelic SNPs under evolutionary constraint by examining sites overlapping phyloP evolutionary constraint scores from the dog-referenced version of the 240 species Cactus alignment (3). We calculated the constraint score cutoffs at various false discovery rates (FDR).

### Unique, rare, and potentially deleterious variants

We first identified all “population-unique” variants, defined as those observed in the representative dog from a population (either once or twice) and not observed in representatives from any of the other populations. With this method, we identified 206,164 population-unique variants for Balto, 120,279 for the Alaskan sled dog, 119,482 variants for the Greenland sled dog, 120,780 unique to the Alaskan malamute, and 133,200 unique to the Siberian husky. We confirmed that population-unique variants tend to be uncommon by calculating the allele frequencies in its population. We used Zoonomia PhyloP scores and SnpEff(42) annotations to identify which population-unique variants were either “evolutionarily constrained” (phyloP score above the FDR 0.05 cutoff of 2.56) or a missense mutation and thus more likely to have functional consequences (table S15). We grouped the dogs into working dog groups including Balto, Alaskan sled dog, and Greenland sled dog, and modern breeds including all the other 54 dogs. We then applied Student’s t-test on the percentage of “evolutionarily constrained” or missense mutation for the two groups.

### Derived, common, and potentially beneficial variants

We identified “homozygous derived” variants, defined as those observed twice in the representative dog from a population and not observed in wolves, for each of the populations. With this method, we identified 176,135 homozygous derived variants for Balto, 148,036 variants for Alaskan sled dog, 260,457 variants for Greenland sled dog, 225,270 variants for Alaskan Malamute, and 189,188 variants for Siberian husky. We confirmed that homozygous variants in each representative dog tend to be “common” in their population by calculating the allele frequency of the homozygous derived variants in its own breed. We also used a Wilcox test against randomly selected SNPs to show that population-unique SNPs are rare, whereas homozygous derived SNPs are rather common, among their population.

We further defined variants likely to be functional as those that were both “highly evolutionarily constrained” (defined by phyloP score above the FDR>0.01 cutoff of 3.52) and a missense mutation. We annotated the variant by genes, and performed gene set enrichment against all Gene Ontology Biological Process gene sets (http://geneontology.org/) using the R package rbioapi v. 0.7.4 (43, 44) (table S7, S8). We also tested for overlap between Balto’s variant genes and genes implicated in particular phenotypes in human studies using the Human Phenotype Ontology (24) and the “Investigate gene sets” feature provided by GSEA (http://www.gsea-msigdb.org/) (table S9).

### Prediction of Balto’s aesthetic phenotypes

We extracted Balto’s genotypes for a panel of 27 genetic variants associated with physical appearance in domestic dogs (table S5) to infer his coat coloration, patterning, and type. We also phased haplotypes from Balto’s genotypes using *EAGLE* (v.2.4.1) (51) with reference haplotypes from the phased UMass-Broad Canid Variants and constructed the haplotype consensus sequences of the *MITF*-M promoter length polymorphism locus (chr20: 21,839,331 – 21,839,366) and upstream SINE insertion locus (chr20: 21,836,232 – 21,836,429) using *BCFtools* in order to investigate the *MITF* variants that putatively affect white spotting. We also ran a body size prediction for Balto using a random forest model (*R* packages `caret` and `randomForest`) built on the relative heights (defined as where a dog’s shoulders fall relative to an “average person”, and surveyed on a Likert scale from ankle-high and shorter, or survey option 0, to hip-high and taller, or survey option 4) of 1,730 modern pet dogs surveyed and 2,797 size-associated SNPs genotyped by the Darwin’s Ark project described previously (12) (see supporting files for model and scripts used to run prediction).

### Balto’s physiological adaptations

We examined the genotypes underlying 14 regions (table S6), which included 1 region under selection in high altitude individuals (53) (Endothelial PAS domain-containing protein 1-*EPAS1*), 2 regions previously identified as under selection in sled dogs (10) (Calcium Voltage-Gated Channel Subunit Alpha1 A - *CACNA1A* and Maltase-Glucoamylase - *MGAM*), 8 regions identified by population branch statistics as potentially under selection in sled dog breeds (12), and 3 regions responsible for aesthetic phenotypes described previously in domestic dogs (Melanocortin 1 Receptor - *MC1R (45)*, Agouti Signaling Protein - *ASIP (52)*, and a chr28 cis-regulatory region associated with single-layered coats (13)). Following the method outlined in Bergström et al. 2020 (21), we also investigated the number of Amylase Alpha 2B (*AMY2B*) copies Balto had by quantifying the ratio of reads (reads/total length of region) mapping to the *AMY2B* regions in CanFam3.1 (ratio: 0.20) to the number of reads mapping to 75 randomly chosen 1kb windows of the genome (ratio: 0.59), given that higher copy numbers are suggested for dog adaptation to starch-rich diets (22).

## Supplementary Material

supplementary tables 1-12

supplementary materials

## Data Availability

Raw sequencing reads for Balto and Alaskan sled dogs have been deposited to the NCBI Sequence Read Archive under BioProject accession PRJNA786530.
