# Supplementary material for: Comparative genomics of Balto, a famous historic dog, captures lost diversity of 1920s sled dogs
Source: Science. Author manuscript; Available in PMC 2023 May 15. (PMC10184777; doi:10.1126/science.abn5887)
Supplement: supplementary materials [file NIHMS1893725-supplement-supplementary_materials.docx]

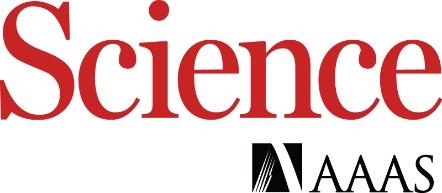


Supplementary Materials for

Comparative genomics of Balto, a famous historic dog, captures lost diversity of 1920s sled dogs

Katherine L. Moon^1,2† *^, Heather J. Huson^3†^, Kathleen Morrill^4,5†^, Ming-Shan Wang^1,2^, Xue Li^4,5^, Krishnamoorthy Srikanth^3^, Gavin Svenson^6^, Zoonomia Consortium, Elinor K. Karlsson^4,5‡^ , Beth Shapiro^1,2‡^

Correspondence to: katielouisemoon@gmail.com (KMM); hjh3@cornell.edu (HJH); kathleen.morrill@umassmed.edu (KM); beth.shapiro@gmail.com (BS); elinor.karlsson@umassmed.edu (EKK).

## This PDF file includes:

Supplementary Text

Materials and Methods

Figures S1 to S8

References (19 - 47)

## Other Supplementary Material for this manuscript includes the following:

Tables S1 to S12

## Supplementary Text

### Balto’s history and records

Balto was bred by renowned sled dog driver, Leonard Seppela, in Nome, Alaska, in 1919. He is by far one of the most famed sled dogs in history, running on the final sled dog relay team to deliver critical antitoxin serum to Nome, Alaska, during the 1925 diphtheria outbreak. Driven by dog musher Gunnar Kaasen, Balto became the media face of all sled dogs contributing to the relay and was credited with their feats of courage and endurance. In an unprecedented moment in history for sled dogs before or thereafter, Kaasen, with Balto and 11 other dogs, toured the U.S. as heroes. A statue of Balto, erected in 1925, resides in New York City’s Central Park and commemorates the indomitable spirit of sled dogs.

Regrettably, the fame of Balto is somewhat shadowed in the eyes of sled dog mushers and the history of mushing. Prior to the Serum Run, records indicate that Balto was used on Seppala’s freight team but not one of his main dogs. He was neutered at six months of age and had no offspring. As such, Balto was left in Nome to support Pioneer Mining Company needs when Seppala embarked with his main team to retrieve the serum. Seppala’s team, who traversed the longest and most treacherous part of the trail, included his well known dogs; Fritz, Billiken, Pete, Young Scotty, and his famed leader, Togo. Due to the epidemic worsening in Nome, more teams were sent out to assist in the relay. This included Gunnar Kaasen driving the team Seppala left in Nome. That team consisted of another of Seppala’s top lead dogs, Fox, as well as Balto. Exactly what role Balto played in Kaasen’s team, whether a lead dog or team dog, he did run the last 53 miles in the successful transport of the serum to Nome (*19*). He brought national fame to sled dogs in 1925 serving as a hero dog and advocate for working sled dogs for almost a century now.

As hype for the Serum Run ebbed, the Cleveland Museum of Natural History took over ownership of Balto and six kennelmates in March of 1927 where they lived out their lives at the Brookside Zoo as working dog heroes (*20*). Balto died at the age of 14, on March 14, 1933. He was subsequently mounted and placed on display within the Museum’s permanent collection. Balto’s DNA was extracted from a piece of stomach tissue obtained from his taxidermied body on display at the Museum.

While Balto’s role and that of other famous Seppala dogs remind people of the Serum Run, their notoriety as Leonard Seppala’s dogs also plays an important role in the history and development of modern breeds such as the Siberian husky and Alaskan malamute and modern working Alaskan sled dogs and Inuit dogs. Leonard Seppala is attributed with starting some of the early lines of working dogs contributing to the modern Siberian husky of today. His dogs were known to be directly descended from early sledding dogs imported from Siberia and mixes of these Siberian dogs and what was considered to be early Alaskan malamutes (*19*). The Inuit dog or Greenland sled dog, and modern racing Alaskan sled dogs are similarly thought to be derived from early Siberian dogs and mixtures of Siberian, malamute, and hound dogs respectively (*6*, *21*). As a Leonard Seppala dog, Balto has a unique genetic tie to modern arctic breed dogs.

From historical photos and taxidermied remains, Balto bore a double-layered coat with extensively dark fur and minimal white spotting on his lip, chest, and legs. His eyes were dark-colored, and while his exact weight and height were not recorded, Balto was regarded as more “stout and strong” than the “small, fast huskies for racing” that his breeder, Leonard Seppala, purportedly produced (*20*).

## Materials and Methods

### Assembly of comparative canid genetic variants

We collated a reference set of comparative canid genetic variants starting from the curated Broad-UMass Canid Variant set, which covers a considerable amount of the extant genetic diversity in *Canis* species derived from whole genome sequencing data in 676 dogs, wolves, coyotes, and other canids, and comprising 34,191,821 single nucleotide polymorphisms (SNPs) and 11,943,064 insertions / deletions (indels) jointly called against the CanFam3.1 reference assembly using the Genome Analysis Toolkit (GATK3, nightly version from June 24th, 2016) for 531 dogs of known breed ancestry distributed among 132 breeds, 28 dogs of mixed breed ancestry, 12 dogs of unknown ancestry, 69 worldwide indigenous or village dogs, 33 wolves, and 1 coyote. The full set of samples used from this collection in subsequent analysis is described in Table S1.

### Ancient DNA extraction, library preparation, and genome assembly

Following his death in 1933, Balto was taxidermied to be exhibited in the Cleveland Museum of Natural History, where he has remained on display behind glass. In order to preserve the specimen, a small piece of his underbelly skin with hair was used for destructive sampling. We processed the sample in a clean lab specifically built for ancient DNA, which excludes PCR products and modern DNA, to prevent contamination. We extracted DNA from a ~5mm x 5mm piece of underbelly skin tissue, in two replicates (HM246 and HM247) with an extraction negative, using the ancient DNA specific protocol in Dabney et al. 2013 (*22*). We cut the tissue into smaller pieces using a sterile scalpel and incubated it on a rotator overnight at 55°C in 0.5mL of extraction buffer (2% w/v SDS; 10mM Tris, pH 8.0; 2.5mM EDTA, pH 8.0; 10mM NaCl; 5mM CaCl 2 ; 0.04M DTT; 0.25 mg/mL of Proteinase K, pH 8.0) which was UV irradiated before use. We then pelleted the tissue in a centrifuge, removed the supernatant carefully and combined it with 13.5mL of UV irradiated binding buffer (4.8M guanidine hydrochloride, 38.5% isopropanol, 0.05% Tween, 111 mM sodium acetate), before forcing it through a MinElute column paired with a Zymo reservoir extender (*22*) in a centrifuge for 10 minutes at 1000 rpm. We dry spun (1 min at 13000 rpm) the sample, washed it twice with 750ul of PE buffer (1 min at 6000 rpm), dry spun it again, incubated it at room temperature for 5 minutes with 2 x 25 ul of EBT and spun it (30 sec at 13000 rpm) to elute the DNA. We quantified the extracts using a Qubit (1X dsDNA HS Assay kit) with 2ul of input.

We prepared 25 ~1pmol input Illumina libraries for extract HM246, 7 for extract HM247 following the Santa Cruz library preparation method (*23*), which is a single-stranded library preparation method specifically designed for ancient samples. For each group of libraries, we also ran a positive control, and a negative control. We quantified all 32 libraries using qPCR (1XMaxima SYBR Green PCR Master Mix, 200 nM IS7, 200 nM IS8, 2uL of 1:100d library), to inform the ideal cycle number for index PCR amplification. We amplified the libraries for 7-8 cycles (1X Amplitaq Gold 360 Master Mix, 2uM i7 Index, 2uM i5 Index) accordingly, and cleaned them using a 1.2X SPRI protocol.

We sequenced all 32 libraries to a depth of ~1 million reads to calculate quality control (QC) statistics including endogenous content, average read length, and complexity. All 32 libraries passed QC, and so we sequenced them to a depth of ~2.3 billion on a NovaSeq 6000 platform 150bp paired end (see Table S9 for the number of reads produced per library). We used SeqPrep v.1.1 (*24*) to trim adapters, remove reads shorter than 28bp, and merge remaining paired-end reads with a minimum overlap of 15 bp.

We then used the Burrows-Wheeler Aligner (BWA) v.0.7.12 (*25*) with a minimum quality cut off of 20 to align reads to the *Canis lupus familiaris* (dog) reference genome (CanFam3.1) (NCBI: GCA_000002285.2). We attached read groups, indicating the library the sequences came from, to each sorted bam file produced by BWA, and all 32 bam files (one for each library) were merged into one with PCR duplicates removed. We used both Qualimap (v2.2.1) and samtools (v1.7) to calculate metrics and assess the quality of the alignment (see Table S10).

### Alaskan sled dogs

Alaskan sled dogs are a recognized mixed breed population of dogs commonly used for competitive racing, recreational sport, and tourism. They are bred specifically for performance in arctic conditions. Working sled dogs of the late 1800’s and early 1900’s commonly traversed longer distances carrying heavy loads providing mail and supply transport. With the advent of modern arctic transportation, the sport of sled dog racing increased as mushers (driver of the team) still sought the title of best dog breeder and driver. In turn, selection of sled dogs over the past century diverged with dogs selected primarily for endurance or speed (*6*). Elite endurance racing encompasses races such as the Iditarod (www.iditarod.com), the 1,000 mile commemorative race of the Serum Run from Anchorage to Nome, Alaska, covering the path the serum antitoxin was transported by both train and dog team in 1925. In contrast, elite sprint teams race two to three days on courses less than 30 miles to win based on a total combined fastest time. Alaskan sled dogs are a genetically unique group of dogs, similarly identifiable by genetic signature as breed dogs. Their intense selection for sprint and distance racing creates genetic substructure within the population. In contrast to breed dogs, Alaskan sled dogs have an open breeding program allowing admixture with any dog of any breed or population and select strictly on performance with no prescribed standards. Despite their unique genomic population signature, traces of Siberian Husky, Alaskan Malamute, Pointer, and Saluki are observed in modern Alaskan sled dogs (*6*). The three Alaskan sled dogs sequenced for this study represented distance sled dogs from kennels competing in races such as the Iditarod. Owner consent was received prior to study commencement and followed an approved animal safety protocol by Cornell University’s Institutional Animal Care and Use Committee (protocol #2014- 0121). Whole blood (3ml) was collected with Ethylenediaminetetraacetic acid (K_2_EDTA) anticoagulant from the cephalic vein. Genomic DNA was extracted following the Gentra Puregene Blood Kit Protocol (Gentra Systems, Inc. Minneapolis, MN, USA) using laboratory-made buffers. Approximately 3μg of DNA was used for library preparation using Illumina TruSeq PCR-free DNA preparation kit (Illumina, San Diego, CA, USA). `AlaskanSledDog_Spot` was sequenced on nextseq 500 (2 x 150bp), while `AlaskanSledDog_Markle` and `AlaskanSledDog_Zorro` were sequenced on NovaSeq 6000 (2 x 150 bp). Mapping to *Canis lupus familiaris* (dog) reference genome (CanFam3.1) was performed using BWA-Mem v.0.7.15 (*25*) using default options.

### Variant calling of Balto, Alaskan sled dogs, and Greenland sled dogs

For Balto as well as 10 previously published Greenland sled dogs (*7*) and the 3 Alaskan sled dogs, we used GATK HaplotypeCaller to call variants in Balto against the UMass-Broad Canid Variant set using parameter *--genotyping-mode* GENOTYPE_GIVEN_ALLELES *--alleles* (known alleles). Then, we merged variant call records from these 14 dogs with records from the UMass-Broad Candid Variants set, for variant calls in a full set of 688 individuals: Balto, 3 modern Alaskan sled dogs, 10 modern Greenland sled dogs, 531 dogs from modern breeds, 40 dogs of unknown or admixed ancestry, 69 village or indigenous dogs, 33 wolves, and 1 coyote.

### Global ancestry inference

We inferred Balto’s ancestral similarity to modern dog breeds, sled dog type breeds, and working sled dogs using a custom built reference panel of contemporary dogs and canids. We selected publicly available genotype data (*N*= 2,166) (*9*, *26*, *27*) (see Table S11) from 109 modern breeds with at least 4 dogs per breed, 2 modern sled dog populations (3 Alaskan sled dogs and 11 Greenland sled dogs), 3 regional village dog populations (4 Nigerian village dogs, 5 Vietnamese village dogs, 55 Chinese village dogs), 3 coyotes, and 2 wolf populations (19 North American wolves and 25 Eurasian wolves). In *PLINK* (v2.00a3LM) (*28*), we identified 4,267,732 biallelic single nucleotide polymorphisms with <10% missing genotypes, and calculated Wright’s F-statistics using Hudson method (*29*, *30*) for (1) each dog breed and sled dog population versus all other dogs; (2) all village dogs versus all other dogs; (3) each regional village dog population; (4) all wolves versus all other dogs; (5) all coyotes versus all other canids; and (6) North American wolves versus Eurasian wolves. We selected 1,858,634 SNPs with *F_ST_*>0.5 across all comparisons, and performed LD-based pruning in 250kb windows for *r^2^*>0.2 to extract 136,779 markers for global ancestry inference.

We merged Balto’s genotypes for these SNPs with genotypes from reference samples, then performed global ancestry inference using *ADMIXTURE (31)* in supervised mode (random seed: 43) with 20 bootstrap replicates to estimate parameter standard errors. We found he shared most of his ancestry with modern Siberian huskies (36.47%) and Alaskan (34.16%) and Greenland (10.45%) sled dogs. Our results also provide evidence for shared ancestry with East Asian dogs in the detection of 12.93% Vietnamese village dog ancestry and 5.87% Tibetan mastiff ancestry.

### Phylogenetic analysis and neighbor-joining trees

In order to place Balto’s phylogenetic relationship to other individuals of *Canis lupus familiaris*, we representatively sampled Balto and a total of 99 other individuals with the highest coverage genomes for each designated population of modern dog breeds (76 breeds, 1 dog/breed), modern sled dog breeds (2 Alaskan malamutes, 1 Siberian husky, and 3 dogs of Siberian husky ancestry), and working sled dogs (3 Alaskan sled dogs and 5 Greenland sled dogs), outbred dogs (1 Chinese indigenous dog and 1 Chinese village dog), as well as 5 wolves, including 2 Grey wolves (*Canis lupus*), a Tibetan wolf (*Canis lupus chanco*), a Iberian wolf (*Canis lupus signatus*), a red wolf (*Canis rufus*), and a coyote (*Canis latrans*) as outgroups (see Table S1 for samples selected in the `Phylogenetic Analysis`).

We confirmed Balto’s phylogenetic position, using all 100 representative canids by generating a neighbor-joining (NJ) phylogenetic tree and conducting a principal component analysis (PCA). We converted the variant calls into a FASTA file and used MEGA-CC(*32*) with 1000 bootstraps to assess tree topology. We ran a PCA on this set using *PLINK* (v1.9), and then visualized the first two principal components in R (v. 3.6.3) using the `ggplot2` package.

### Homozygosity and inbreeding metrics

From the 100 representative individuals, we further removed any sample with any (>0.00%) missing genotypes, leaving 86 individuals (see Table S1 for samples selected in the `Homozygosity Analysis`). We detected runs of homozygosity (RoH) using a window-based approach implemented in *PLINK* (v1.9) (*28*). We applied the parameters outlined in Foote et al. 2021 (*33*): a window size of 300kb, and allowing up to 3 heterozygous sites in each window, with a requirement of 1000kb between two SNPs to be considered in different RoH, and with a minimum of 50 SNPs at a minimum density of 1 SNP per 50kb required to call a RoH. We calculated two measures of inbreeding: the method-of-moments coefficient in *PLINK* (*F_MoM_*) and the metric based on runs-of-homozygosity (*F_RoH_*), as recommended by Zhao et al. 2020 (*34*) (Table S3). *F_RoH_* is a function of genomic coverage by RoH, which correlates strongly with pedigree-based inbreeding metrics (*35*), and *F_MoM_* is the probability of inheriting two identical by descent alleles at an autosomal loci, dependent on population-estimated allele frequencies. Using the *R* (v. 3.6.3) function `cor.test`, we confirmed that *F_RoH_* and *F_MoM_* are significantly correlated (*R*_Pearson_= 0.6752819, *p*= 9.958e-13, *t*= 8.3913, *df*= 84).

| **Breed** | **N** | **Number of RoH segments** | **Average size of RoH (kb)** | **F(RoH)** | **F(MoM)** |
| --- | --- | --- | --- | --- | --- |
| Alaskan sled dogs | 3 | 142-237 | 421-435 | 0.03-0.05 | 0.10-0.17 |
| **Balto** | **1** | **105** | **376** | **0.02** | **0.14** |
| Greenland sled dogs | 5 | 114-328 | 370-415 | 0.02-0.06 | 0.36-0.43 |
| Sled dog breeds | 3 | 637-774 | 491-523 | 0.15-0.17 | 0.15-0.24 |
| Dogs from other modern breeds | 73 | 220-1620 | 396-699 | 0.04-0.44 | -0.02-0.57 |

### Population representative sampling

As Balto is the sole representative of his contemporary population, we randomly selected one representative sample from each of 57 populations for the discovery of individually-represented, population-relevant genetic variants (see Table S1 for samples selected in the `Population Variants Analysis`) among 67,085,518 biallelic single nucleotide polymorphisms, and included Balto, 1 Alaskan sled dog, 1 Greenland sled dog, and 54 modern purebred dogs, including 1 Siberian husky and 1 Alaskan malamute. Likewise, we selected, where available, another 5 to 11 random samples from 10 modern breeds, and all remaining Greenland sled dog samples, to assess the population-wide allele frequency of these variants (see Table S1 `Population Frequency Analysis`).

### Dog-referenced mammalian evolutionary constraint

We selected biallelic SNPs under evolutionary constraint by examining sites overlapping phyloP evolutionary constraint scores from the dog-referenced version of the 240 species Cactus alignment (*1*). We calculated the constraint score cutoffs at various false discovery rates (FDR).

|  | **phyloP cutoff** | **Percentage of Genome (total: 2.33Gb)** | **Bases (Mb)** |
| --- | --- | --- | --- |
| < 1% FDR | 3.52 | 2.11% | 49.2 |
| < 3% FDR | 2.88 | 2.84% | 66.0 |
| < 5% FDR | 2.56 | 3.43% | 79.8 |
| < 10% FDR | 2.1 | 4.69% | 109.2 |
| < 20% FDR | 1.59 | 7.02% | 163.4 |

### Unique, rare, and deleterious variants

We first identified all “population-unique” variants, defined as those observed in the representative dog from a population (either once or twice) and not observed in representatives from any of the other populations. With this method, we identified 206,164 population-unique variants for Balto, 120,279 for the Alaskan sled dog, 119,482 variants for the Greenland sled dog, 120,780 unique to the Alaskan malamute, and 133,200 unique to the Siberian husky.

We hypothesized that Balto, and dogs from modern working sled dog populations, should carry fewer rare, damaging genetic variants than modern, pedigreed sled dog breeds selected primarily for aesthetics (Siberian husky and Alaskan malamute). We confirmed that population-unique variants tend to be uncommon by calculating the allele frequencies in its population.

| **Frequencies of population-unique variants** | | | | |
| --- | --- | --- | --- | --- |
| **Breed** | **# dogs** | **# variants** | **MAF <0.1** | **MAF <0.2** |
| Bearded Collie | 7 | 39,305 | 0.205 | 0.320 |
| Belgian Sheepdog | 7 | 32,289 | 0.296 | 0.520 |
| Belgian Tervuren | 11 | 32,467 | 0.366 | 0.650 |
| Bernese Mountain Dog | 8 | 36,267 | 0.183 | 0.419 |
| Border Collie | 5 | 46,966 | 0.307 | 0.590 |
| Bull Terrier | 6 | 35,459 | 0.256 | 0.395 |
| Entlebucher Sennenhund | 8 | 38,647 | 0.073 | 0.224 |
| German Shepherd | 11 | 48,457 | 0.401 | 0.558 |
| Greenland Sled Dog | 7 | 119,481 | 0.304 | 0.470 |
| Labrador Retriever | 10 | 45,138 | 0.381 | 0.690 |
| Tibetan Mastiff | 11 | 239,374 | 0.400 | 0.736 |

We applied this on ten breeds and one working dog group: bearded collie, Belgian sheepdog, Belgian Tervuren, Bernese mountain dog, Border Collie, Bull terrier, Entlebucher Sennenhund, German shepherd dog, Greenland sled dog, Labrador retriever, and Tibetan mastiff, each with 5 to 11 dogs. We used Zoonomia PhyloP scores and SNPeff annotations to identify which population-unique variants were either “evolutionarily constrained” (phyloP score above the FDR 0.05 cutoff of 2.56) or a missense mutation (determined using SnpEff(*36*)), and thus more likely to have functional consequences (Table S12). We grouped the dogs into working dog groups including Balto, Alaskan Sled dog, and Greenland Sled dog, and modern breeds including all the other 54 dogs. We then applied Student’s t-test on the percentage of “evolutionarily constrained” or missense mutation for the two groups.

### Derived, common, and potentially beneficial variants

We identified “homozygous derived” variants, defined as those observed twice in the representative dog from a population and not observed in wolves, for each of the populations. With this method, we identified 176,135 homozygous derived variants for Balto, 148,036 variants for Alaskan sled dog, 260,457 variants for Greenland sled dog, 225,270 variants for Alaskan Malamute, and 189,188 variants for Siberian husky. We confirmed that homozygous variants in each representative dog tend to be “common” in their population by calculating the allele frequency of the homozygous derived variants in its own breed using the same set of dogs described above for calculating allele frequency for population-unique variants.

| **Frequencies of derived homozygous variants** | | | | |
| --- | --- | --- | --- | --- |
| **Breed** | **# dogs** | **# variants** | **MAF >0.2** | **MAF >0.5** |
| Bearded Collie | 7 | 232,077 | 0.995 | 0.846 |
| Belgian Sheepdog | 7 | 215,483 | 0.973 | 0.722 |
| Belgian Tervuren | 11 | 218,736 | 0.927 | 0.676 |
| Bernese Mountain Dog | 8 | 239,978 | 0.969 | 0.816 |
| Border Collie | 5 | 192,661 | 1.000 | 0.792 |
| Bull Terrier | 6 | 291,487 | 0.993 | 0.907 |
| Entlebucher Sennenhund | 8 | 260,140 | 0.997 | 0.905 |
| German Shepherd | 11 | 218,212 | 0.926 | 0.718 |
| Greenland Sled Dog | 7 | 260,456 | 0.969 | 0.795 |
| Labrador Retriever | 10 | 218,402 | 0.958 | 0.618 |
| Tibetan Mastiff | 11 | 146,749 | 0.933 | 0.514 |

We additionally show that population-unique SNPs are rare, whereas homozygous derived SNPs are rather common, among their population using a Wilcox test against randomly selected SNPs.

|  |  | **Allele Frequency in Population** | | | | | | **Wilcox test (vs all)** | |
| --- | --- | --- | --- | --- | --- | --- | --- | --- | --- |
| **Class** | **# variants** | **mean** | **median** | **Q25** | **Q75** | **min** | **max** | **W** | ***p*** |
| randomly sampled | 275,846 | 0.451 | 0.375 | 0.167 | 0.714 | 0.045 | 1 |  |  |
| unique in one dog representing population | 713,850 | 0.247 | 0.182 | 0.091 | 0.333 | 0.045 | 1 | 61956762263 | <2.2e-16 |
| derived & homozygous | 2,494,381 | 0.677 | 0.700 | 0.500 | 0.909 | 0.091 | 1 | 487126516909 | <2.2e-16 |

We further defined variants likely to be functional as those that were both “highly evolutionarily constrained” (defined by phyloP score above the FDR>0.05 cutoff of 3.63) and a missense mutation. We annotated the variant by genes, and performed gene set enrichment against all Gene Ontology Biological Process gene sets (http://geneontology.org/) using the R package rbioapi v. 0.7.4(*37*)(Table S6). We tested for overlap between Balto’s variant genes and genes implicated in particular phenotypes in human studies using the Human Phenotype Ontology(*38*) and the “Investigate gene sets'' feature provided by GSEA (http://www.gsea-msigdb.org/).

### Prediction of Balto’s aesthetic phenotypes

We extracted Balto’s genotypes for a panel of 27 genetic variants associated with physical appearance in domestic dogs (Table S4) to infer his coat coloration, patterning, and type. He carried none of the derived alleles associated with single-layered coats in dogs, so he would presumably have the ancestral double-layered coat. Canine coat coloration varies according to the expression of two pigmentation proteins: eumelanin, a dark pigment that expresses as brown or black (*39*–*41*), and pheomelanin, a light pigment ranging from cream to red (*42*, *43*). Balto carried none of the eumelanin alleles associated with brown pigmentation (*40*, *44*), five intensity-associated alleles that predict expression of a lighter shade of pheomelanin (*15*) He was homozygous for an insertion variant responsible for a black-and-tan coat pattern(*12*) but had a dominant allele known to cause a melanistic facial mask (*14*) that, in combination with his lighter pheomelanin pigmentation, would probably have masked the tan points in his coat. Notably, Balto lacked any alleles for Northern domino (*16*), a coat pattern often seen in Siberian huskies, although his melanistic facial mask would have overridden its expression as well. Balto also carries one copy of the allele associated with blue eyes in modern Siberian huskies (*13*), which is unlikely to affect his eye color, as dogs with this marker that also express a melanistic mask are significantly more likely to have brown, rather than blue, eyes.

We phased haplotypes from Balto’s genotypes using *EAGLE* (v.2.4.1) (*45*) with reference haplotypes from the phased UMass-Broad Canid Variants. For the *MITF* variants that putatively affect white spotting, we constructed the haplotyped consensus sequences of the *MITF*-M promoter length polymorphism locus (chr20: 21,839,331 - 21,839,366) and upstream SINE insertion locus (chr20: 21,836,232 - 21,836,429) using *BCFtools*. Balto carries a pair of shorter of length polymorphism alleles, C_11_A_9_G_2_A_12_ (32bp) / C_10_A_9_G_2_A_12_ (31bp) (chr20: 21,839,331 - 21,839,366), more similar to solidly colored breeds, although he is homozygous for the SINEC-Cf insertion (chr20: 21,836,232 - 21,836,429), which is not found in solid-colored breeds, and he lacks the A>G SNP at chr5: 21,838,204.

We also ran a body size prediction for Balto using a random forest model built on the relative heights (defined as where a dog’s shoulders fall relative to an “average person”, and surveyed on a Likert scale from ankle-high and shorter, or survey option 0, to hip-high and taller, or survey option 4) of 1,730 modern pet dogs surveyed and 2,797 size-associated SNPs genotyped by the Darwin’s Ark project. We predicted that Balto would stand around 55 cm tall at the shoulder (predicted value: 2.3 on survey scale), which is on the small end of modern, pedigreed sled dogs from the Darwin’s Ark study (purebred Siberian huskies surveying on average 2.6 ± SD: 0.6, and purebred Alaskan malamutes surveying on average 3.0 ± SD: 0.5) (Fig. S8). In agreement with this prediction, photos of Balto as an adult dog show his shoulders at approximately knee height of the people with whom he is posed.

### Balto’s physiological adaptations

We examined the genotypes underlying 14 regions (Table S5), which included 1 region under selection in high altitude individuals (*EPAS1*), 2 regions previously identified as under selection in sled dogs (*CACNA1A* and *MGAM*), 8 regions identified by population branch statistics as potentially under selection in sled dog breeds, and 3 regions responsible for aesthetic phenotypes described previously in domestic dogs (*MC1R(39)*, *ASIP(46)*, and a chr28 cis-regulatory region associated with single-layered coats (*10*)). We found no evidence that Balto was adapted to the hypoxic conditions of high altitudes, as he did not carry the haplotype of *EPAS1* found in the Tibetan mastiff and the Chinese indigenous dog (*47*). Contrary to the expectation for sled dogs (*7*), we found evidence of adaptation to starch-rich diets in Alaskan sled dogs and in Balto. Greenland sled dogs and wolves are known to carry an ancestral *MGAM* haplotype at high frequency (*7*), and so we expected Balto and the Alaskan sled dogs to follow suit. Instead, Alaskan sled dogs predominantly had the dog-specific haplotype (frequency of 83%), and Balto carried a copy each of the ancestral and dog-specific copy. Given the dog-specific *MGAM* haplotype is associated with an ability to digest starch (*17*), this suggests the isolation of Greenland sled dogs, who had the haplotype at frequency of only 20%, has prevented their adaptation to starch-rich diets that occurred in dogs elsewhere. Other regions lacked sufficient bootstrap support to infer phylogenetic relationships.

## Supplementary Figures


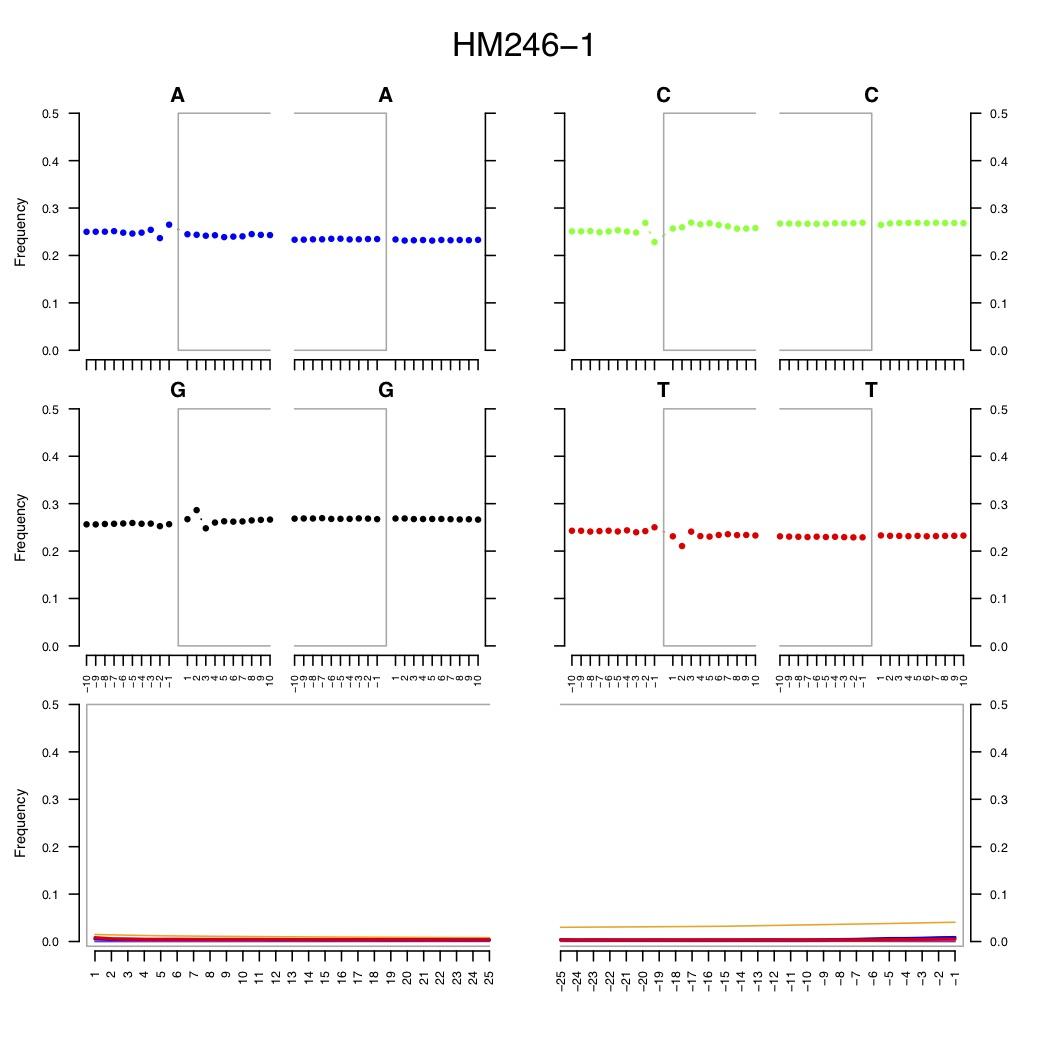


**Fig. S1.** Damage plot of sequencing library HM246 showing low (<1%) rates of damage from mapped reads. While most ancient samples have higher rates of damage at the ends of sequenced reads, Balto’s DNA was well preserved. Other aspects of his data (e.g. short reads; lack of contamination in controls) support the authenticity of these data.


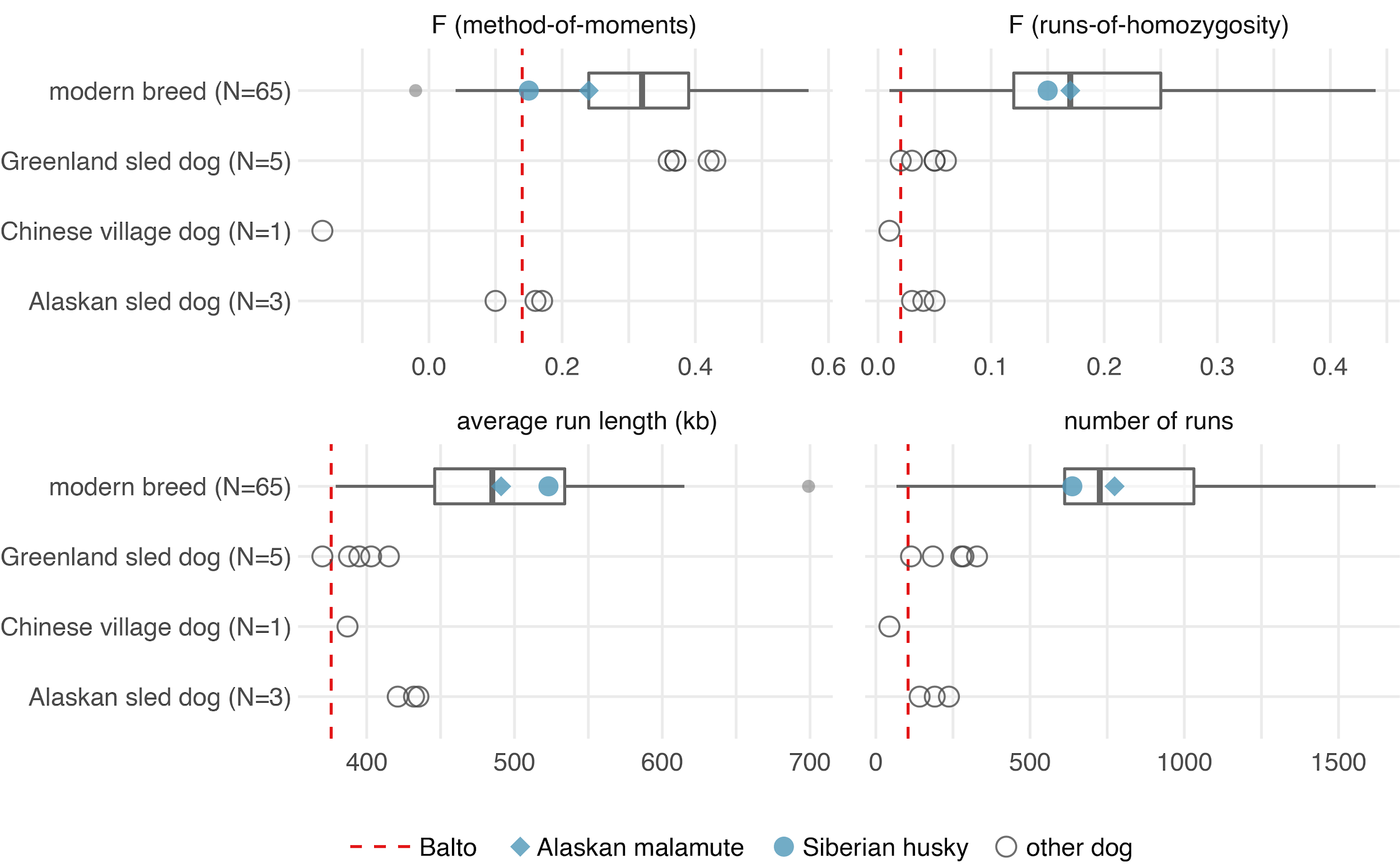


**Fig. S2.** For 65 breed-representative dogs, 5 Greenland sled dogs, 3 Alaskan sled dogs, and 1 Chinese village dog, we detected runs of homozygosity, estimated inbreeding *F_RoH_* from autosomal coverage of runs, and calculated the method-of-moments *F_MoM_* coefficient of inbreeding from heterozygosity. Balto and modern Alaskan sled dogs had lower inbreeding by both metrics than most modern breeds, including Alaskan malamute and Siberian husky, but Greenland sled dogs had exceptionally high homozygosity.

#### **Fig. S3.** The allele frequencies, across breeds and populations, of randomly sampled, “common” (derived homozygous) and “rare” (unique in representative dog) single-nucleotide polymorphisms.
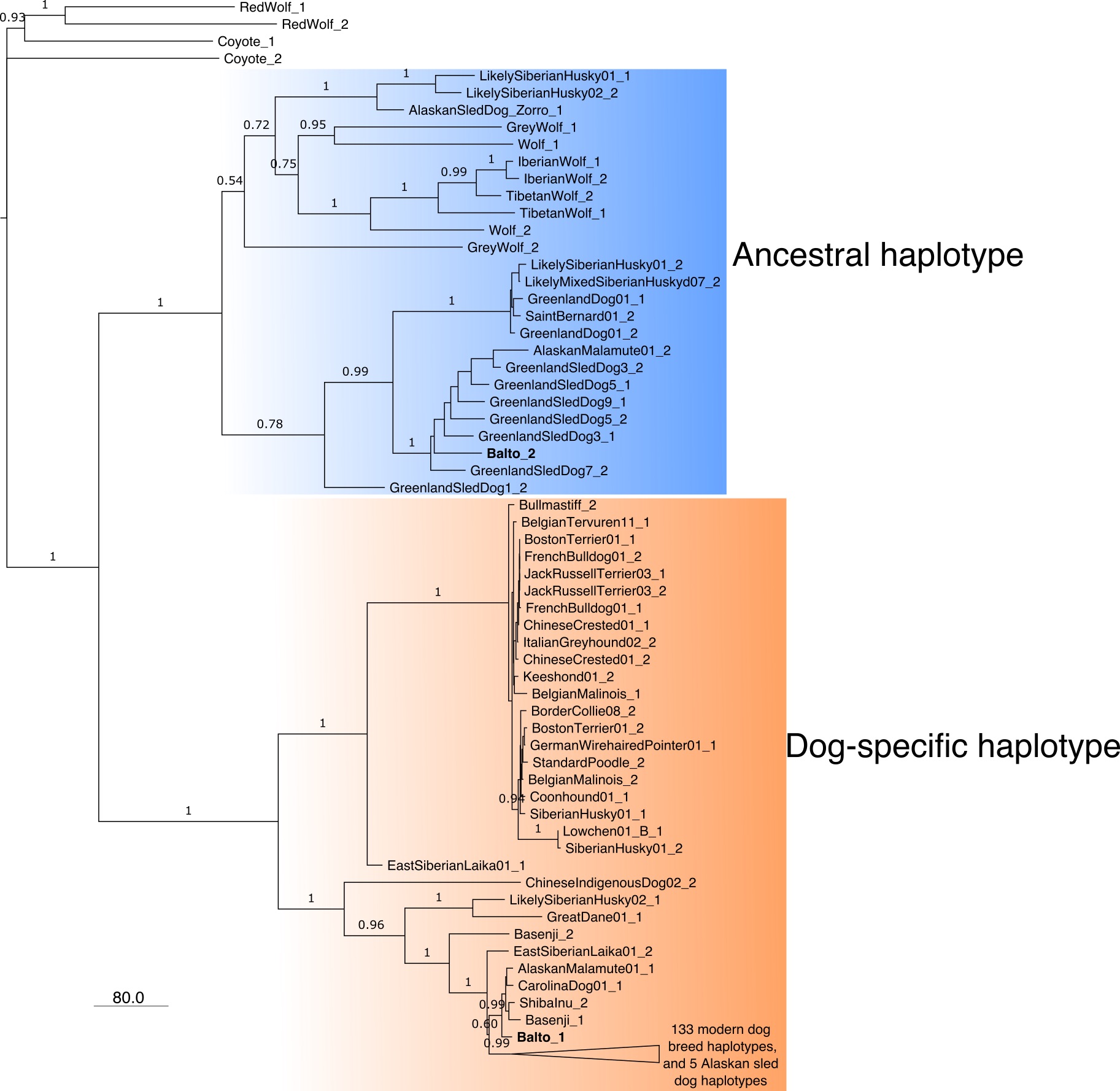


#### **Fig. S4.** Phylogenetic tree of *MGAM* gene region from 100 dogs with support values (to 2 dp) based on 1,000 bootstraps of the data. Balto (**bold**) had one ancestral copy (blue) and one modern dog-specific (orange) copy.


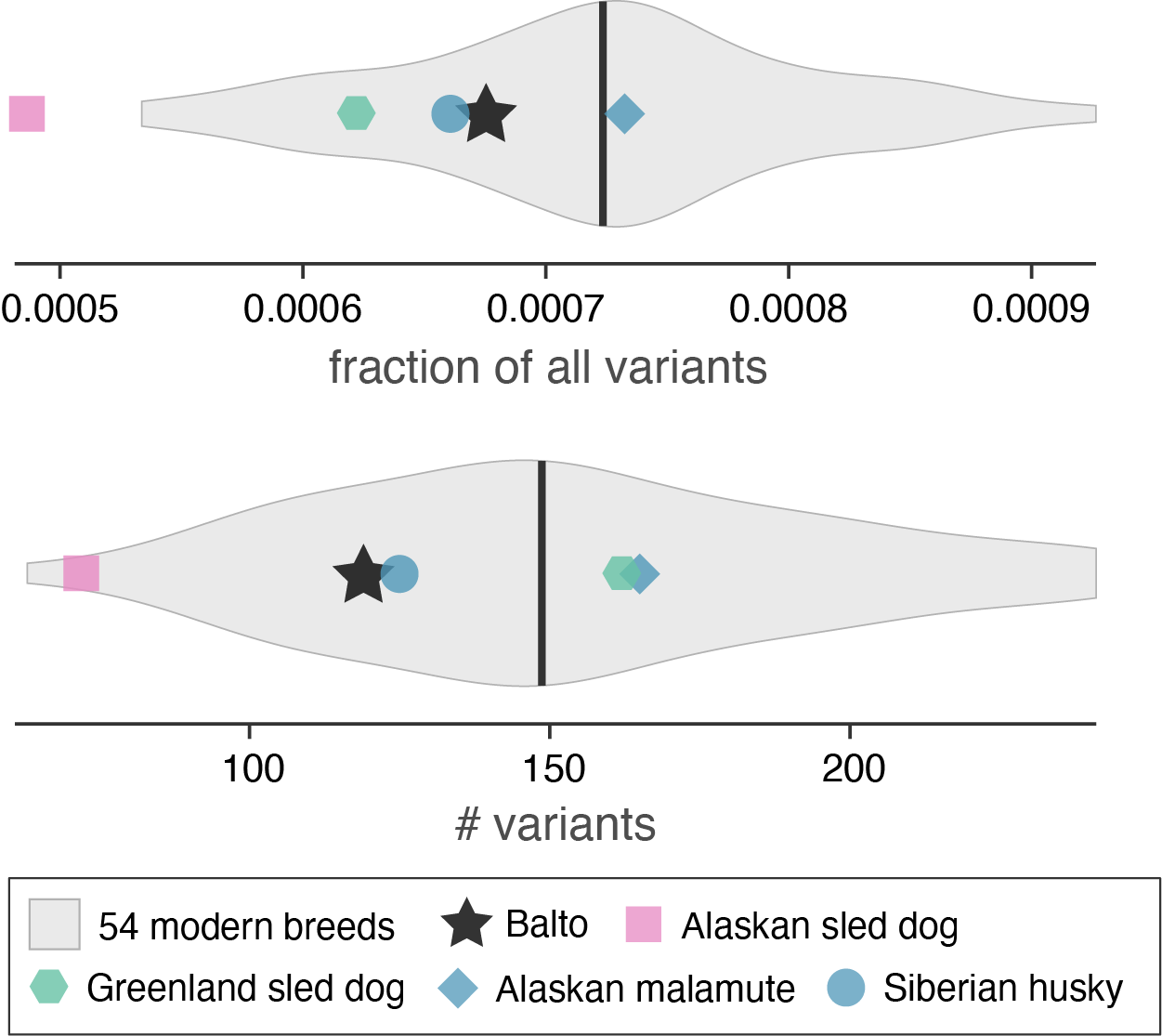


**Fig. S5.**  The distribution of missense and highly evolutionarily constrained (FDR<0.01) single nucleotide polymorphisms in single dogs representing modern breeds, working sled dogs and in Balto.


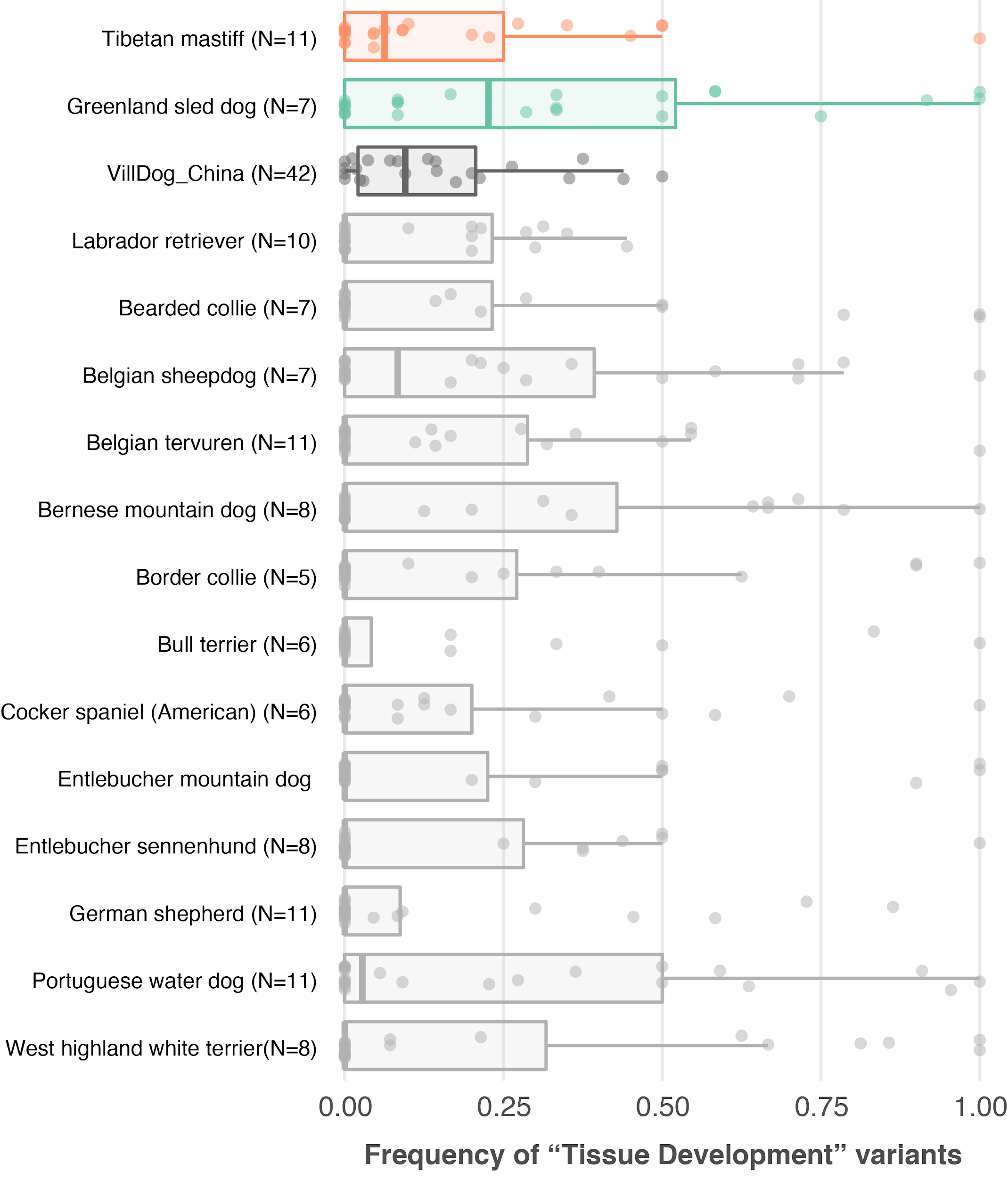


**Fig. S6.** The frequency of Balto variants in genes belonging to the “tissue development” ontology term across populations, including populations with which Balto shares ancestry such as the Tibetan mastiff (orange), Greenland sled dogs (green), and East asian village dogs (dark grey) and other breeds (light grey). Only populations with 5 or more sequenced dogs are shown.


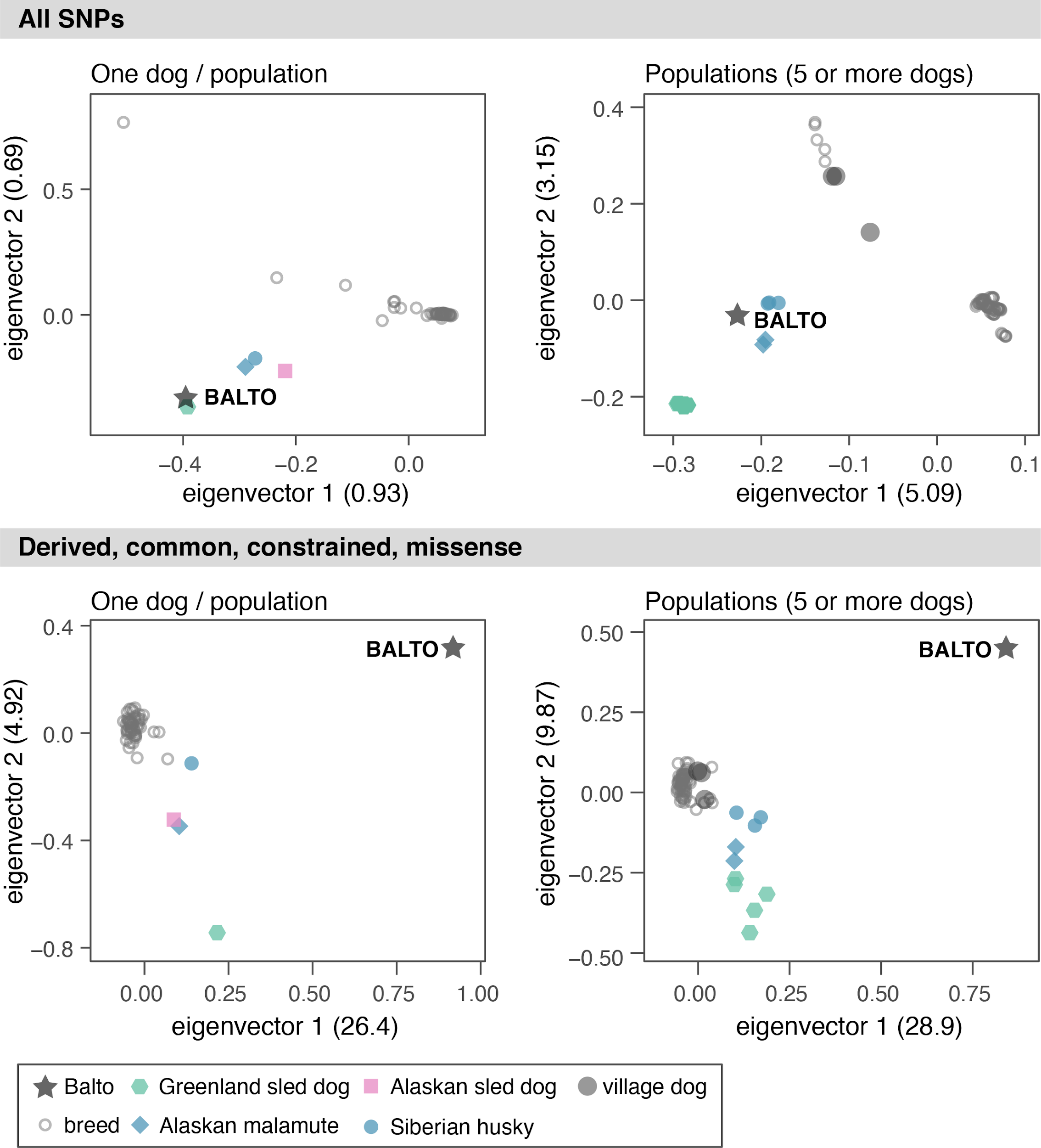


**Fig. S7.** Principal component analysis of representative dogs and their populations along all single nucleotide polymorphisms (SNPs) and those SNPs that are derived (non-wolf), common (homozygous in representative dog), constrained (phyloP selected for FDR <1%) and missense (predicted by SnpEff), for which Balto was separated out even from other sled dogs and sled dog breeds.


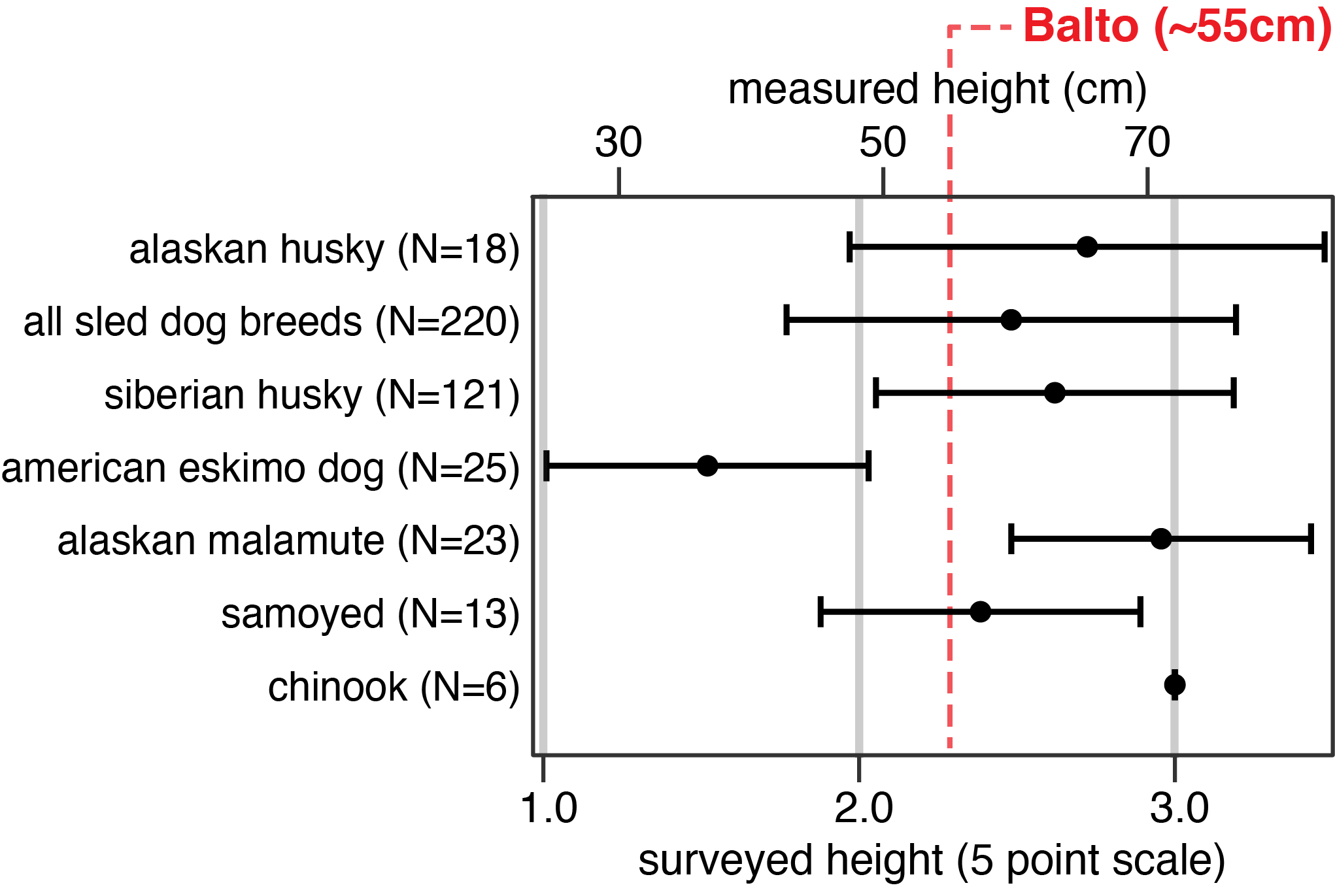


**Fig. S8.** Using a random forest model based on 1,730 pet dogs and 2,797 height-associated genetic variants, we predicted that Balto would stand around 55 cm tall (value: 2.3) at his withers, placing Balto within the heights surveyed for sled dog breeds like modern, purebred Siberian huskies (average value 2.6 ± SD: 0.6).

## References and Notes

20. M. J. Christmas, I. M. Kaplow, D. P. Genereux, M. X. Dong, G. M. Hughes, X. Li, P. F. Sullivan, A. G. Hindle, G. Andrews, J. C. Armstrong, M. Bianchi, A. M. Breit, M. Diekhans, C. Fanter, N. M. Foley, L. Goodman, K. C. Keough, B. Kirilenko, A. Kowalczyk, C. Lawless, A. Lind, J. R. S. Meadows, L. Moreira, L. Ryan, R. Swofford, A. Valenzuela, F. Wagner, O. Wallerman, J. Damas, K. Fan, J. Grimshaw, J. Johnson, S. V. Kozyrev, A. J. Lawler, V. D. Marinescu, A. Osmanski, N. S. Paulat, B. N. Phan, S. K. Reilly, D. E. Schäffer, C. Steiner, M. A. Supple, A. P. Wilder, M. E. Wirthlin, J. R. Xue, B. W. Birren, S. Gazal, R. M. Hubley, K.-P. Koepfli, T. Marques-Bonet, W. Meyer, M. Nweeia, B. Shapiro, A. F. A. Smit, M. Springer, E. Teeling, Z. Weng, M. Hiller, D. L. Levesque, H. Lewin, W. J. Murphy, A. Navarro, B. Paten, K. S. Pollard, D. A. Ray, I. Ruf, O. A. Ryder, A. R. Pfenning, K. Lindblad-Toh, E. K. Karlsson, Evolutionary constraint and innovation across hundreds of placental mammals. *Science*.

2. J. Meadows, S. Gazal, P. Sullivan, Zoonomia Consortium, E. K. Karlsson, K. Lindblad-Toh, Leveraging Base Pair Mammalian Constraint to Understand Genetic Variation and Human Disease. *Science*.

3. G. Salisbury, L. Salisbury, *The Cruelest Miles: The Heroic Story of Dogs and Men in a Race Against an Epidemic* (W. W. Norton & Company, 2003; https://play.google.com/store/books/details?id=0oJiGYk-QVcC).

4. N. B. Sutter, D. S. Mosher, M. M. Gray, E. A. Ostrander, Morphometrics within dog breeds are highly reproducible and dispute Rensch’s rule. *Mamm. Genome*. **19**, 713–723 (2008).

5. American Kennel Club, *The Complete Dog Book: 20th Edition* (Random House Publishing Group, 2007; https://play.google.com/store/books/details?id=NqXaduT6Ak0C).

6. H. J. Huson, H. G. Parker, J. Runstadler, E. A. Ostrander, A genetic dissection of breed composition and performance enhancement in the Alaskan sled dog. *BMC Genet.* **11**, 71 (2010).

7. M.-H. S. Sinding, S. Gopalakrishnan, J. Ramos-Madrigal, M. de Manuel, V. V. Pitulko, L. Kuderna, T. R. Feuerborn, L. A. F. Frantz, F. G. Vieira, J. Niemann, J. A. Samaniego Castruita, C. Carøe, E. U. Andersen-Ranberg, P. D. Jordan, E. Y. Pavlova, P. A. Nikolskiy, A. K. Kasparov, V. V. Ivanova, E. Willerslev, P. Skoglund, M. Fredholm, S. E. Wennerberg, M. P. Heide-Jørgensen, R. Dietz, C. Sonne, M. Meldgaard, L. Dalén, G. Larson, B. Petersen, T. Sicheritz-Pontén, L. Bachmann, Ø. Wiig, T. Marques-Bonet, A. J. Hansen, M. T. P. Gilbert, Arctic-adapted dogs emerged at the Pleistocene–Holocene transition. *Science*. **368**, 1495–1499 (2020).

8. A. V. Shindyapina, A. A. Zenin, A. E. Tarkhov, D. Santesmasses, P. O. Fedichev, V. N. Gladyshev, Germline burden of rare damaging variants negatively affects human healthspan and lifespan. *Elife*. **9** (2020), doi:10.7554/eLife.53449.

9. K. Morrill, J. Hekman, X. Li, J. McClure, B. Logan, L. Goodman, M. Gao, Y. Dong, M. Alonso, E. Carmichael, N. Snyder-Mackler, J. Alonso, H. J. Noh, J. Johnson, M. Koltookian, C. Lieu, K. Megquier, R. Swofford, J. Turner-Maier, M. E. White, Z. Weng, A. Colubri, D. P. Genereux, K. A. Lord, E. K. Karlsson, Ancestry-inclusive dog genomics challenges popular breed stereotypes. *Science*.

10. D. T. Whitaker, E. A. Ostrander, Hair of the Dog: Identification of a Cis-Regulatory Module Predicted to Influence Canine Coat Composition. *Genes* . **10** (2019), doi:10.3390/genes10050323.

11. E. K. Karlsson, I. Baranowska, C. M. Wade, N. H. C. Salmon Hillbertz, M. C. Zody, N. Anderson, T. M. Biagi, N. Patterson, G. R. Pielberg, E. J. Kulbokas 3rd, K. E. Comstock, E. T. Keller, J. P. Mesirov, H. von Euler, O. Kämpe, A. Hedhammar, E. S. Lander, G. Andersson, L. Andersson, K. Lindblad-Toh, Efficient mapping of mendelian traits in dogs through genome-wide association. *Nat. Genet.* **39**, 1321–1328 (2007).

12. D. L. Dreger, H. G. Parker, E. A. Ostrander, S. M. Schmutz, Identification of a mutation that is associated with the saddle tan and black-and-tan phenotypes in Basset Hounds and Pembroke Welsh Corgis. *J. Hered.* **104**, 399–406 (2013).

13. P. E. Deane-Coe, E. T. Chu, A. Slavney, A. R. Boyko, A. J. Sams, Direct-to-consumer DNA testing of 6,000 dogs reveals 98.6-kb duplication associated with blue eyes and heterochromia in Siberian Huskies. *PLoS Genet.* **14**, e1007648 (2018).

14. S. M. Schmutz, T. G. Berryere, N. M. Ellinwood, J. A. Kerns, G. S. Barsh, MC1R studies in dogs with melanistic mask or brindle patterns. *J. Hered.* **94**, 69–73 (2003).

15. A. J. Slavney, T. Kawakami, M. K. Jensen, T. C. Nelson, A. J. Sams, A. R. Boyko, Five genetic variants explain over 70% of hair coat pheomelanin intensity variation in purebred and mixed breed domestic dogs. *PLoS One*. **16**, e0250579 (2021).

16. H. Anderson, L. Honkanen, P. Ruotanen, J. Mathlin, J. Donner, Comprehensive genetic testing combined with citizen science reveals a recently characterized ancient MC1R mutation associated with partial recessive red phenotypes in dog. *Canine Med Genet*. **7**, 16 (2020).

17. E. Axelsson, A. Ratnakumar, M.-L. Arendt, K. Maqbool, M. T. Webster, M. Perloski, O. Liberg, J. M. Arnemo, A. Hedhammar, K. Lindblad-Toh, The genomic signature of dog domestication reveals adaptation to a starch-rich diet. *Nature*. **495**, 360–364 (2013).

18. X. Gou, Z. Wang, N. Li, F. Qiu, Z. Xu, D. Yan, S. Yang, J. Jia, X. Kong, Z. Wei, S. Lu, L. Lian, C. Wu, X. Wang, G. Li, T. Ma, Q. Jiang, X. Zhao, J. Yang, B. Liu, D. Wei, H. Li, J. Yang, Y. Yan, G. Zhao, X. Dong, M. Li, W. Deng, J. Leng, C. Wei, C. Wang, H. Mao, H. Zhang, G. Ding, Y. Li, Whole-genome sequencing of six dog breeds from continuous altitudes reveals adaptation to high-altitude hypoxia. *Genome Res.* **24**, 1308–1315 (2014).

19. B. Thomas, P. Thomas, *Leonhard Seppala: The Siberian Dog and the Golden Age of Sleddog Racing 1908-1941* (Pictorial Histories Publishing Company, Incorporated, 2015; https://play.google.com/store/books/details?id=Tx1qjwEACAAJ).

20. The Cleveland Museum of Natural History, Balto FAQs, (available at https://www.cmnh.org/science-news/blog/march-2020/balto-faq).

21. Sled dog central: The Inuit sled dog by sue Hamilton, (available at http://www.sleddogcentral.com/inuit.htm).

22. J. Dabney, M. Knapp, I. Glocke, M.-T. Gansauge, A. Weihmann, B. Nickel, C. Valdiosera, N. García, S. Pääbo, J.-L. Arsuaga, M. Meyer, Complete mitochondrial genome sequence of a Middle Pleistocene cave bear reconstructed from ultrashort DNA fragments. *Proc. Natl. Acad. Sci. U. S. A.* **110**, 15758–15763 (2013).

23. J. D. Kapp, R. E. Green, B. Shapiro, A Fast and Efficient Single-stranded Genomic Library Preparation Method Optimized for Ancient DNA. *J. Hered.* **112**, 241–249 (2021).

24. J. S. John, SeqPrep: tool for stripping adaptors and/or merging paired reads with overlap into single reads. *URL: https://githubcom/jstjohn/SeqPrep* (2011).

25. H. Li, R. Durbin, Fast and accurate long-read alignment with Burrows–Wheeler transform. *Bioinformatics*. **26**, 589–595 (2010).

26. J. J. Hayward, M. G. Castelhano, K. C. Oliveira, E. Corey, C. Balkman, T. L. Baxter, M. L. Casal, S. A. Center, M. Fang, S. J. Garrison, S. E. Kalla, P. Korniliev, M. I. Kotlikoff, N. S. Moise, L. M. Shannon, K. W. Simpson, N. B. Sutter, R. J. Todhunter, A. R. Boyko, Complex disease and phenotype mapping in the domestic dog. *Nat. Commun.* **7**, 10460 (2016).

27. J. Plassais, J. Kim, B. W. Davis, D. M. Karyadi, A. N. Hogan, A. C. Harris, B. Decker, H. G. Parker, E. A. Ostrander, Whole genome sequencing of canids reveals genomic regions under selection and variants influencing morphology. *Nat. Commun.* **10**, 1489 (2019).

28. S. Purcell, B. Neale, K. Todd-Brown, L. Thomas, M. A. R. Ferreira, D. Bender, J. Maller, P. Sklar, P. I. W. de Bakker, M. J. Daly, P. C. Sham, PLINK: a tool set for whole-genome association and population-based linkage analyses. *Am. J. Hum. Genet.* **81**, 559–575 (2007).

29. B. S. Weir, C. C. Cockerham, ESTIMATING F-STATISTICS FOR THE ANALYSIS OF POPULATION STRUCTURE. *Evolution*. **38**, 1358–1370 (1984).

30. G. Bhatia, N. Patterson, S. Sankararaman, A. L. Price, Estimating and interpreting FST: the impact of rare variants. *Genome Res.* **23**, 1514–1521 (2013).

31. D. H. Alexander, K. Lange, Enhancements to the ADMIXTURE algorithm for individual ancestry estimation. *BMC Bioinformatics*. **12**, 246 (2011).

32. S. Kumar, G. Stecher, D. Peterson, K. Tamura, MEGA-CC: computing core of molecular evolutionary genetics analysis program for automated and iterative data analysis. *Bioinformatics*. **28**, 2685–2686 (2012).

33. A. D. Foote, R. Hooper, A. Alexander, R. W. Baird, C. S. Baker, L. Ballance, J. Barlow, A. Brownlow, T. Collins, R. Constantine, L. Dalla Rosa, N. J. Davison, J. W. Durban, R. Esteban, L. Excoffier, S. L. F. Martin, K. A. Forney, T. Gerrodette, M. T. P. Gilbert, C. Guinet, M. B. Hanson, S. Li, M. D. Martin, K. M. Robertson, F. I. P. Samarra, R. de Stephanis, S. B. Tavares, P. Tixier, J. A. Totterdell, P. Wade, J. B. W. Wolf, G. Fan, Y. Zhang, P. A. Morin, Runs of homozygosity in killer whale genomes provide a global record of demographic histories. *Mol. Ecol.* (2021), doi:10.1111/mec.16137.

34. G. Zhao, T. Zhang, Y. Liu, Z. Wang, L. Xu, B. Zhu, X. Gao, L. Zhang, H. Gao, G. E. Liu, J. Li, L. Xu, Genome-Wide Assessment of Runs of Homozygosity in Chinese Wagyu Beef Cattle. *Animals (Basel)*. **10** (2020), doi:10.3390/ani10081425.

35. R. McQuillan, A.-L. Leutenegger, R. Abdel-Rahman, C. S. Franklin, M. Pericic, L. Barac-Lauc, N. Smolej-Narancic, B. Janicijevic, O. Polasek, A. Tenesa, A. K. Macleod, S. M. Farrington, P. Rudan, C. Hayward, V. Vitart, I. Rudan, S. H. Wild, M. G. Dunlop, A. F. Wright, H. Campbell, J. F. Wilson, Runs of homozygosity in European populations. *Am. J. Hum. Genet.* **83**, 359–372 (2008).

36. P. Cingolani, snpEff: Variant effect prediction (2012).

37. M. Rezwani, rbioapi: User-Friendly R Interface to Biologic Web Services’ API (2021), (available at https://CRAN.R-project.org/package=rbioapi).

38. S. Köhler, M. Gargano, N. Matentzoglu, L. C. Carmody, D. Lewis-Smith, N. A. Vasilevsky, D. Danis, G. Balagura, G. Baynam, A. M. Brower, T. J. Callahan, C. G. Chute, J. L. Est, P. D. Galer, S. Ganesan, M. Griese, M. Haimel, J. Pazmandi, M. Hanauer, N. L. Harris, M. J. Hartnett, M. Hastreiter, F. Hauck, Y. He, T. Jeske, H. Kearney, G. Kindle, C. Klein, K. Knoflach, R. Krause, D. Lagorce, J. A. McMurry, J. A. Miller, M. C. Munoz-Torres, R. L. Peters, C. K. Rapp, A. M. Rath, S. A. Rind, A. Z. Rosenberg, M. M. Segal, M. G. Seidel, D. Smedley, T. Talmy, Y. Thomas, S. A. Wiafe, J. Xian, Z. Yüksel, I. Helbig, C. J. Mungall, M. A. Haendel, P. N. Robinson, The Human Phenotype Ontology in 2021. *Nucleic Acids Res.* **49**, D1207–D1217 (2021).

39. S. M. Schmutz, T. G. Berryere, A. D. Goldfinch, TYRP1 and MC1R genotypes and their effects on coat color in dogs. *Mamm. Genome*. **13**, 380–387 (2002).

40. E. J. Cargill, T. R. Famula, R. D. Schnabel, G. M. Strain, K. E. Murphy, The color of a Dalmatian’s spots: linkage evidence to support the TYRP1 gene. *BMC Vet. Res.* **1**, 1 (2005).

41. S. Kiener, A. Kehl, R. Loechel, I. Langbein-Detsch, E. Müller, D. Bannasch, V. Jagannathan, T. Leeb, Novel Brown Coat Color (Cocoa) in French Bulldogs Results from a Nonsense Variant in HPS3. *Genes* . **11** (2020), doi:10.3390/genes11060636.

42. J. A. Kerns, J. Newton, T. G. Berryere, E. M. Rubin, J.-F. Cheng, S. M. Schmutz, G. S. Barsh, Characterization of the dog Agouti gene and a nonagouti mutation in German Shepherd Dogs. *Mamm. Genome*. **15**, 798–808 (2004).

43. J. A. Kerns, E. J. Cargill, L. A. Clark, S. I. Candille, T. G. Berryere, M. Olivier, G. Lust, R. J. Todhunter, S. M. Schmutz, K. E. Murphy, G. S. Barsh, Linkage and segregation analysis of black and brindle coat color in domestic dogs. *Genetics*. **176**, 1679–1689 (2007).

44. L. V. Monteagudo, M. T. Tejedor, The b(c) allele of TYRP1 is causative for the recessive brown (liver) colour in German Shepherd dogs. *Anim. Genet.* **46**, 588–589 (2015).

45. P.-R. Loh, P. Danecek, P. F. Palamara, C. Fuchsberger, Y. A Reshef, H. K Finucane, S. Schoenherr, L. Forer, S. McCarthy, G. R. Abecasis, R. Durbin, A. L Price, Reference-based phasing using the Haplotype Reference Consortium panel. *Nat. Genet.* **48**, 1443–1448 (2016).

46. T. G. Berryere, J. A. Kerns, G. S. Barsh, S. M. Schmutz, Association of an Agouti allele with fawn or sable coat color in domestic dogs. *Mamm. Genome*. **16**, 262–272 (2005).

47. B. vonHoldt, Z. Fan, D. Ortega-Del Vecchyo, R. K. Wayne, EPAS1 variants in high altitude Tibetan wolves were selectively introgressed into highland dogs. *PeerJ*. **5**, e3522 (2017).
